# Engineered Probiotic‐Based Personalized Cancer Vaccine Potentiates Antitumor Immunity through Initiating Trained Immunity

**DOI:** 10.1002/advs.202305081

**Published:** 2023-11-27

**Authors:** Zhaoxia Chen, Tuying Yong, Zhaohan Wei, Xiaoqiong Zhang, Xin Li, Jiaqi Qin, Jianye Li, Jun Hu, Xiangliang Yang, Lu Gan

**Affiliations:** ^1^ National Engineering Research Center for Nanomedicine College of Life Science and Technology Huazhong University of Science and Technology Wuhan 430074 China; ^2^ Key Laboratory of Molecular Biophysics of the Ministry of Education College of Life Science and Technology Huazhong University of Science and Technology Wuhan 430074 China; ^3^ Hubei Key Laboratory of Bioinorganic Chemistry and Materia Medica Huazhong University of Science and Technology Wuhan 430074 China

**Keywords:** antitumor immunity, β‐glucan, cancer vaccines, probiotics, trained immunity

## Abstract

Cancer vaccines hold great potential for clinical cancer treatment by eliciting T cell‐mediated immunity. However, the limited numbers of antigen‐presenting cells (APCs) at the injection sites, the insufficient tumor antigen phagocytosis by APCs, and the presence of a strong tumor immunosuppressive microenvironment severely compromise the efficacy of cancer vaccines. Trained innate immunity may promote tumor antigen‐specific adaptive immunity. Here, a personalized cancer vaccine is developed by engineering the inactivated probiotic *Escherichia coli* Nissle 1917 to load tumor antigens and β‐glucan, a trained immunity inducer. After subcutaneous injection, the cancer vaccine delivering model antigen OVA (BG/OVA@EcN) is highly accumulated and phagocytosed by macrophages at the injection sites to induce trained immunity. The trained macrophages may recruit dendritic cells (DCs) to facilitate BG/OVA@EcN phagocytosis and the subsequent DC maturation and T cell activation. In addition, BG/OVA@EcN remarkably enhances the circulating trained monocytes/macrophages, promoting differentiation into M1‐like macrophages in tumor tissues. BG/OVA@EcN generates strong prophylactic and therapeutic efficacy to inhibit tumor growth by inducing potent adaptive antitumor immunity and long‐term immune memory. Importantly, the cancer vaccine delivering autologous tumor antigens efficiently prevents postoperative tumor recurrence. This platform offers a facile translatable strategy to efficiently integrate trained immunity and adaptive immunity for personalized cancer immunotherapy.

## Graphical abstract

A personalized cancer vaccine is developed by engineering the inactivated probiotic *Escherichia coli* Nissle 1917 to load tumor antigens, and β‐glucan (BG/OVA@EcN). BG/OVA@EcN efficiently induces trained immunity in macrophages to enhance adaptive immunity. Meanwhile, BG/OVA@EcN‐augmented circulating trained monocytes/macrophages promote differentiation into M1‐like macrophages in tumor tissues, generating strong prophylactic and therapeutic efficacy to inhibit tumor growth.

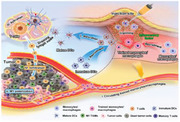

## 1 Introduction

Cancer immunotherapy that boosts the host immune response to kill tumor cells has shown major clinical benefits in cancer patients.^[^ ^1^ ^]^ Among the various cancer immunotherapies, cancer vaccines, which depend on tumor antigens and adjuvants to promote antigen presentation by antigen‐presenting cells (APCs) and activate the adaptive antitumor immune response and immune memory, are emerging as a promising approach to kill tumor cells and inhibit tumor recurrence and metastasis.^[^ ^2^ ^]^ However, the limited numbers of APCs at the injection sites,^[^ ^3^ ^]^ the insufficient tumor antigen phagocytosis and presentation by APCs,^[^ ^4^ ^]^ and the presence of strong tumor immunosuppressive microenvironment,^[^ ^5^ ^]^ such as tumor‐associated macrophages (TAMs) substantially compromise the efficacy of cancer vaccines. Thus, efficiently recruiting APCs, increasing tumor antigen phagocytosis and presentation by APCs, and improving tumor immunosuppressive microenvironment are required to increase the therapeutic outcomes of cancer vaccines.

Innate immune cells are critical to antitumor immunosurveillance by functioning as a rapid first line of defense through the recognition of pathogens and endogenous danger signals.^[^ ^6^ ^]^ Although immune memory is a defining feature of the acquired immune cells, emerging evidence has shown that these innate immune cells possess a form of immune memory termed trained immunity.^[^ ^6^, ^7^ ^]^ These trained immune cells undergo transcriptomic, epigenetic, and metabolic reprogramming upon specific initial stimulation, and remain more responsive to the secondary heterologous stimulus in an enhanced inflammatory response.^[^ ^8^ ^]^ Studies have reported that β‐glucan (BG),^[^ ^9^ ^]^ Bacillus Calmette–Guérin,^[^ ^10^ ^]^ and oxidized low‐density lipoprotein,^[^ ^11^ ^]^ can directly stimulate innate immune cells to induce trained immunity. The trained innate immunity plays an important role in antitumor immunotherapy. For example, the trained macrophages exhibit a strong capacity to phagocytose tumor cells, thus efficiently inhibiting tumor growth.^[^ ^9^, ^10^, ^12^ ^]^ Meanwhile, the trained immunity might efficiently enhance adaptive immunity. The trained innate immune cells can release more interleukin‐1β (IL‐1β), IL‐6, and tumor necrosis factor α (TNF‐α), recruiting APCs like dendritic cells (DCs) and promoting antigen phagocytosis^[^ ^13^ ^]^ and presentation to enhance antigen‐specific T cell activation.^[^ ^7^, ^14^ ^]^ In addition, trained immunity can induce bone marrows to produce a large number of trained monocytes/macrophages and increase the proportion of circulating proinflammatory monocytes/macrophages,^[^ ^10^, ^15^ ^]^ resulting in enhanced tumor‐accumulated classically activated monocytes/macrophages and improved tumor immunosuppressive microenvironment.^[^ ^9^, ^15^, ^16^ ^]^ Therefore, the development of cancer vaccines that effectively integrate trained immunity and adaptive immunity to achieve synergistic antitumor immune responses is expected to improve cancer treatment efficacy.

**Figure 1:**
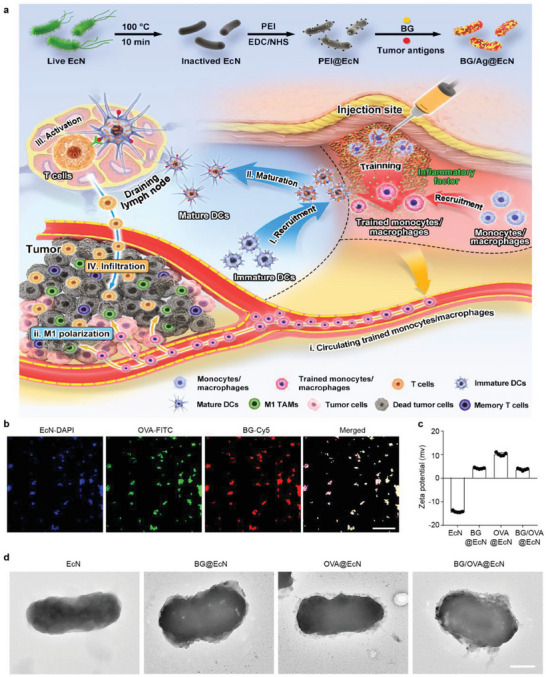
Characterization of BG/OVA@EcN. a) Schematic illustration of BG/Ag@EcN as an efficient cancer vaccine to trigger antitumor immunity for enhanced cancer therapy. BG/Ag@EcN was obtained by modification of inactivated EcN with PEI and then loading BG and tumor antigens. After subcutaneous injection, the macrophages at the injection sites efficiently phagocytose BG/Ag@EcN to induce trained immunity. The trained macrophages secret proinflammatory cytokines to recruit monocytes/macrophages for further training by BG/Ag@EcN. Meanwhile, I) the proinflammatory cytokines secreted by the trained monocytes/macrophages efficiently recruit DCs, II) promoting DC phagocytosis of BG/Ag@EcN for DC maturation, III) T cell activation, and IV) T cell infiltration into tumor tissues. In addition, i) BG/OVA@EcN significantly increases the circulating trained monocytes/macrophages, resulting in ii) differentiating into M1‐like TAMs and improved tumor immunosuppressive microenvironment. b) Confocal microscopic images of BG/OVA@EcN in which BG was conjugated with Cy5, OVA was conjugated with FITC, and EcN was labeled with DAPI. Scale bar: 30 µm. c) Zeta potential of EcN, BG@EcN, OVA@EcN, and BG/OVA@EcN by DLS analysis, respectively. Data are presented as mean ± SD (*n* = 5). d) Representative TEM images of EcN, BG@EcN, OVA@EcN, and BG/OVA@EcN. Scale bar: 0.5 µm.

The use of bacteria has attracted more attention for their potential use in cancer vaccines.^[^ ^17^ ^]^ Bacteria possess pathogen‐associated molecular patterns (PAMPs), such as flagellin, lipopolysaccharide (LPS), peptidoglycan, and so on, exhibiting unique advantages in activating the immune response as innate immune adjuvants.^[^ ^18^ ^]^ In addition, some bacteria have been reported to act as trained immunity inducers.^[^ ^19^ ^]^ For example, *Lactobacillus plantarum* efficiently induced trained immunity to protect against viral infections.^[^ ^20^ ^]^ However, bacteria‐based cancer vaccines exhibited limited therapeutic efficacy.^[^ ^21^ ^]^ Te development of bacteria‐based cancer vaccines with trained innate immunity will enhance adaptive immunity to improve anticancer activity. BG, a structural constituent of cell walls of fungi, has immunomodulatory activity due to their selective recognition by pattern recognition receptors such as dectin‐1, Toll‐like receptors, and complement receptor 3 expressed mainly on immune cells, including macrophages and DCs.^[^ ^22^ ^]^ BG not only as an adjuvant significantly enhances the uptake and presentation of antigens by DCs to promote DC maturation and T cell response,^[^ ^23^ ^]^ but also as a trained immunity inducer of innate immune cells triggers epigenetic changes on histones to promote proinflammatory cytokine production.^[^ ^9a^ ^]^ Here, considering that dead bacterial have several advantages compared to live bacteria, including no virulence pathogenic factors, good safety, easy storage and so on,^[^ ^24^ ^]^ the inactivated *Escherichia coli* Nissle 1917 (EcN), an extensively used probiotic in clinical practice,^[^ ^25^ ^]^ is modified with polyethyleneimine (PEI) and then loaded with BG derived from *Trametes versicolor* (≈100 KDa) and tumor antigens to construct the personalized cancer vaccine. The cancer vaccine delivering model antigen OVA (BG/OVA@EcN) efficiently induces trained immunity of macrophages at the subcutaneous injection sites, secreting high levels of proinflammatory cytokines to promote the recruitment of DCs and the subsequent antigen phagocytosis and cross‐presentation by DCs for improved antitumor immunity. In addition, BG/OVA@EcN significantly enhances the trained monocytes/macrophages in peripheral blood to differentiate into antitumor macrophages in tumor tissues, reshaping the tumor immunosuppressive microenvironment. Thus, BG/OVA@EcN elicits strong adaptive antitumor immunity and immune memory to suppress tumor growth (**Figure** **1**a). Meanwhile, the cancer vaccine delivering autologous tumor antigens efficiently prevents postoperative tumor recurrence by inducing trained innate immunity and immune memory.

## 2 Results

### 2.1 Preparation and Characterization of BG/OVA@EcN

Bacteria and their derived substances can mobilize the immune system to respond to foreign “danger signals” through the innate immune response.^[^ ^17b^ ^]^ To construct a bacteria‐based tumor vaccine, live EcN was first inactivated with high temperature (denoted as EcN) and then modified with PEI to obtain positively charged EcN (denoted as PEI@EcN, Figure S1, Supporting Information). 4,6‐Diamidino‐2‐penylindole (DAPI) and propidium iodide (PI) double staining analysis confirmed that both the inactivated EcN by high temperature and PEI‐modified EcN had almost completely lost their viability (Figure S2, Supporting Information). PEI@EcN was then incubated with BG or/and OVA, a model antigen to obtain BG@EcN, OVA@EcN, or BG/OVA@EcN, respectively. The preparation condition of BG/OVA@EcN was optimized as 1.5 × 10^9^ colony‐forming units (CFU) of EcN mL^−1^, 200 µg mL^−1^ BG, and 200 µg mL^−1^ OVA according to the drug loading capacity (Figure S3a,b, Supporting Information), zeta potential (Figure S3c, Supporting Information), and phagocytosis by DCs (Figure S3d, Supporting Information). The colocalization of fluorescein isothiocyanate (FITC)‐conjugated OVA, Cy5‐labeled BG, and DAPI‐labeled EcN revealed the successful loading of OVA and BG to PEI@EcN in BG/OVA@EcN (Figure 1b). Dynamic light scattering (DLS) analysis showed that the zeta potential of PEI@EcN, BG@EcN, OVA@EcN, and BG/OVA@EcN were 11.6 ±0.5, 4.2 ± 0.2, 10.2 ± 0.6, and 3.7± 0.4, respectively (Figure 1c), demonstrating that the negatively charged BG and OVA were absorbed to PEI@EcN through the electrostatic interaction. Transmission electron microscopy (TEM) showed that BG@EcN, OVA@EcN, and BG/OVA@EcN had a rough surface that was distinct from EcN, further confirming the successful construction of BG/OVA@EcN (Figure 1d). The loading contents of BG and OVA in BG/OVA@EcN were calculated to be about 40 and 40 µg per 3 × 10^8^ colony forming units (CFU) of bacteria, suggesting that BG and OVA were completely loaded into EcN. In addition, BG/OVA@EcN showed high stability in phosphate‐buffered saline (PBS), with no remarkable change in zeta potential within 7 days (Figure S4, Supporting Information). No significant toxicity of BG/OVA@EcN was detected in murine DC2.4 cells (Figure S5a, Supporting Information), RAW264.7 macrophages (Figure S5b, Supporting Information), mouse NIH/3T3 embryonic fibroblasts (Figure S5c, Supporting Information), and human umbilical vein endothelial cells (HUVECs) (Figure S5d, Supporting Information), suggesting its good biocompatibility.

### 2.2 BG/OVA@EcN Efficiently Induces Trained Immunity in Macrophages In Vitro

**Figure 2:**
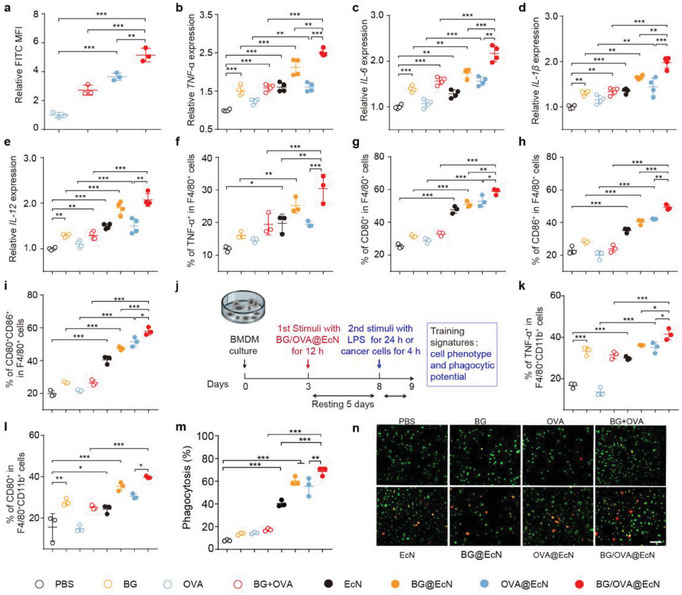
BG/OVA@EcN‐mediated training of monocytes/macrophages in vitro. a) Relative FITC mean fluorescence intensity (MFI) in RAW264.7 cells after treatment with free OVA, BG + OVA, OVA@EcN, or BG/OVA@EcN (OVA was conjugated with FITC) at the concentration of 3 × 10^7^ CFU mL^‐1^ EcN, 4 µg mL^‐1^ OVA, and 4 µg mL^‐1^ BG for 4 h by flow cytometry. Data are presented as mean ± SD (*n* = 3). b–e) mRNA expression levels of b) *TNF‐α*, c) *IL‐6*, d) *IL‐1β*, and e) *IL‐12* in BMDMs after treatment with PBS, BG, OVA, BG + OVA, EcN, BG@EcN, OVA@EcN, and BG/OVA@EcN at the concentration of 3 × 10^7^ CFU mL^‐1^ EcN, 4 µg mL^‐1^ OVA, and 4 µg mL^‐1^ BG for 12 h by real‐time RT‐PCR. Data are presented as mean ± SD (*n* = 4). f–i) Percentages of f) TNF‐α^+^, g) CD80^+^, h) CD86^+^, and i) CD80^+^CD86^+^ cells in F4/80^+^ cells after BMDMs were treated as indicated in (b) by flow cytometry. Data are presented as mean ± SD (*n* = 3). j) Schematic schedule of in vitro BMDM training by BG/OVA@EcN. k) Percentages of TNF‐α^+^ and l) CD80^+^ cells in F4/80^+^CD11b^+^ cells after BMDMs were treated with PBS, BG, OVA, BG + OVA, EcN, BG@EcN, OVA@EcN, and BG/OVA@EcN at the concentration of 3 × 10^7^ CFU mL^−1^ EcN, 4 µg mL^−1^ OVA, and 4 µg mL^‐1^ BG for 12 h, followed by resting for 5 days and re‐stimulating with 100 ng mL^−1^ LPS for 24 h by flow cytometry. Data are presented as mean ± SD (*n* = 3). m) Phagocytosis ratios and n) representative images of DiR‐labeled B16‐OVA cells by CSFE‐labeled BMDMs after BMDMs were treated as indicated in (j) for 12 h, followed by resting for 5 days and then co‐culturing with DiR‐labeled B16‐OVA cells at the ratio of 1:1 for 4 h by flow cytometry and confocal microscopy, respectively. Scale bar: 100 µm. Data are presented as mean ± SD (*n* = 3). *p* values are calculated using one‐way ANOVA followed by Tukey's HSD post‐hoc test. * *p* < 0.05, ** *p* < 0.01, *** *p* < 0.001.

As a pathogen, bacteria can be quickly phagocytosed by APCs.^[^ ^18c^ ^]^ Moreover, it has been well documented that BG as a well‐known PAMP can specifically interact with dectin‐1, mainly expressed in innate immune cells, including macrophages and DCs.^[^ ^22^ ^]^ Expectedly, both OVA@EcN and BG/OVA@EcN showed enhanced phagocytosis by RAW264.7 macrophages compared with OVA alone (**Figure** **2**a and Figure S6, Supporting Information). However, the strongest phagocytosis was detected in the BG/OVA@EcN‐treated group (Figure 2a and Figure S6, Supporting Information).

In view that BG was an inducer of trained immunity, the trained immunity‐inducing potential of BG/OVA@EcN was evaluated in murine bone marrow‐derived macrophages (BMDMs). BMDMs were treated with PBS, BG, OVA, BG combined with OVA (BG + OVA), EcN, BG@EcN, OVA@EcN, or BG/OVA@EcN, and the expressions of proinflammatory molecules, the key markers of trained immunity were determined by real‐time reverse transcription‐polymerase chain reaction (RT‐PCR) (Figure 2b–e). As expected, BG significantly increased the expressions of TNF‐α, IL‐6, IL‐1β, and IL‐12. No significant difference in the expressions of these cytokines was observed in the OVA‐treated group. EcN and OVA@EcN also increased the expressions of these proinflammatory cytokines, which might be due to the existence of PAMPs on EcN. BG@EcN and BG/OVA@EcN demonstrated a stronger ability to promote the expressions of these proinflammatory cytokines, suggesting that BG/OVA@EcN might efficiently induce trained immunity in macrophages. Moreover, the activation markers associated with proinflammatory macrophages were assessed in BMDMs after treatment by flow cytometry. Consistently, BG/OVA@EcN significantly upregulated the ratios of TNF‐α^+^ (Figure 2f), CD80^+^ (Figure 2g), CD86^+^ (Figure 2h), and CD80^+^CD86^+^ macrophages (Figure 2i) compared with other groups, further revealing that BG/OVA@EcN efficiently activated the proinflammatory macrophages.

It has been reported that the innate immune cells can be “trained” to be more responsive to the secondary heterologous stimulus with an enhanced inflammatory response.^[^ ^11^ ^]^ To further confirm whether BMDMs following BG/OVA@EcN treatment displayed a phenotype of trained immunity, a standard training protocol was used in which BMDMs were pretreated with PBS, BG, OVA, BG + OVA, EcN, BG@EcN, OVA@EcN, or BG/OVA@EcN for 12 h, followed by 5 days of rest and then re‐stimulating with LPS for 24 h for determination of ratios of TNF‐α^+^ (a surrogate marker to evaluate the trained immunity) and CD80^+^ macrophages by flow cytometry (Figure 2j–l). As expected, treatment with BG, EcN, or OVA@EcN significantly enhanced the ratios of TNF‐α^+^ and CD80^+^ macrophages compared with the PBS group, verifying that BG and EcN could function as the trained immunity inducers. BG@EcN and BG/OVA@EcN exhibited stronger capacity to promote the ratios of TNF‐α^+^ and CD80^+^ macrophages, suggesting that BG/OVA@EcN efficiently induced trained immunity in macrophages. BG/OVA@EcN‐induced trained immunity was further confirmed in human monocytic THP‐1 cells, as evidenced by the highest ratio of TNF‐α^+^ cells in BG/OVA@EcN‐treated THP‐1 cells compared with other groups (Figure S7, Supporting Information). The trained inflammatory macrophages are shown to have high phagocytic potential. Consistently, the stronger phagocytosis of OVA‐expressing B16 (B16‐OVA) cells was detected in BG/OVA@EcN‐trained BMDMs compared with BG‐ and OVA@EcN‐treated groups by flow cytometry (Figure 2m and Figure S8, Supporting Information). The stronger phagocytosis of B16‐OVA cells by BG/OVA@EcN‐trained BMDMs was further verified by the more colocalization of BG/OVA@EcN‐trained BMDMs and B16‐OVA cells using confocal microscopy (Figure 2n), demonstrating that BG/OVA@EcN could efficiently elicit trained immunity in macrophages. Meanwhile, BG/OVA@EcN‐trained BMDMs exhibited the strongest phagocytosis of B16F10 cells (Figure S9, Supporting Information), suggesting that the BG/OVA@EcN‐induced trained immunity was not antigen‐specific.

### 2.3 BG/OVA@EcN Efficiently Promotes the Recruitment of Monocytes/Macrophages and DCs at the Injection Sites

**Figure 3:**
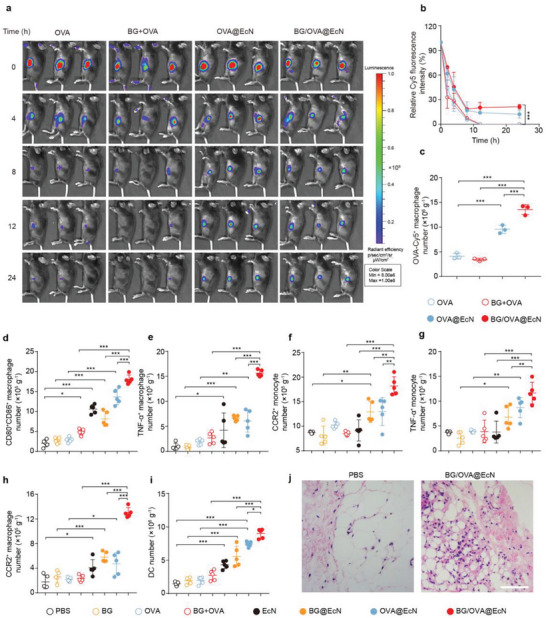
Monocyte/macrophage recruitment and activation induced by BG/OVA@EcN at the injection sites. a) Images and b) relative Cy5 fluorescence intensity at different time intervals after C57BL/6 mice were subcutaneously injected with OVA, BG + OVA, OVA@EcN, or BG/OVA@EcN (OVA was conjugated with Cy5) at the OVA dosage of 40 µg, BG dosage of 40 µg, and EcN dosage of 3 × 10^8^ CFU per mouse. Data are presented as mean ± SD (*n* = 3). c) Percentages of OVA‐Cy5^+^ cells in CD11b^+^F4/80^+^ cells at the injection sites of C57BL/6 mice at 24 h after treatment indicated in (a). Data are presented as mean ± SD (*n* = 3). d–i) Numbers of d) CD80^+^CD86^+^ macrophages, e) TNF‐α^+^ macrophages, f) CCR2^+^ monocytes, g) TNF‐α^+^ monocytes, h) CCR2^+^ macrophages, and i) total DCs (CD11c^+^CD11b^+^CD45^+^) at the injection sites of C57BL/6 mice at 3 days after subcutaneous injection of PBS, BG, OVA, BG + OVA, EcN, BG@EcN, OVA@EcN, or BG/OVA@EcN at the OVA dosage of 40 µg, BG dosage of 40 µg, and EcN dosage of 3 × 10^8^ CFU per mouse. Data are presented as mean ± SD (*n* = 5). j) H&E staining of injection sites of C57BL/6 mice at 3 days after subcutaneous injection of PBS or BG/OVA@EcN at the OVA dosage of 40 µg, BG dosage of 40 µg, and EcN dosage of 3 × 10^8^ CFU per mouse. Scale bar: 25 µm. *p‐*values are calculated using one‐way ANOVA followed by Tukey's HSD post hoc test. * *p* < 0.05, ** *p* < 0.01, *** *p* < 0.001.

After subcutaneous injection of Cy5‐labeled OVA, BG + OVA, OVA@EcN, or BG/OVA@EcN into healthy C57BL/6 mice, OVA in the presence and absence of BG became almost undetectable at 12 h after administration (**Figure** **3**a,b). However, significant Cy5 fluorescence was observed at the injection sites of OVA@EcN‐ and BG/OVA@EcN‐treated groups even after 24 h administration (Figure 3a,b), suggesting that BG/OVA@EcN exhibited a persistent antigen deposition at the injection sites. Consistently, macrophages at the injection sites phagocytosed the most BG/OVA@EcN, about 3.3 and 1.4 times higher than OVA and OVA@ECN, respectively (Figure 3c).

To further determine the immune activation responses of BG/OVA@EcN at the injection sites, healthy C57BL/6 mice were subcutaneously injected with PBS, BG, OVA, BG + OVA, EcN, BG@EcN, OVA@EcN, or BG/OVA@EcN. As expected, treatment with EcN, OVA@EcN, or BG@EcN significantly increased the numbers of CD80^+^CD86^+^ macrophages (CD80^+^CD86^+^F4/80^+^CD11b^+^CD45^+^ cells, Figure 3d), and TNF‐α^+^ macrophages (TNF‐α^+^F4/80^+^CD11b^+^CD45^+^ cells, Figure 3e). BG/OVA@EcN resulted in the highest numbers of CD80^+^CD86^+^ (Figure 3d) and TNF‐α^+^ macrophages (Figure 3e), suggesting that BG/OVA@EcN was efficiently phagocytosed by macrophages to activate the proinflammatory responses at the injection sites. CCR2 is critical for the recruitment of monocytes/macrophages. Meanwhile, the numbers of migratory monocytes (CCR2^+^Ly6C^+^Ly6G^‐^CD11b^+^CD45^+^ cells, Figure 3f), inflammatory monocytes (TNF‐α^+^Ly6C^+^Ly6G^‐^CD11b^+^CD45^+^ cells, Figure 3g), migratory macrophages (CCR2^+^F4/80^+^CD11b^+^CD45^+^ cells, Figure 3h) and total DCs (CD11b^+^CD11c^+^CD45^+^ cells, Figure 3i) were the highest in BG/OVA@EcN‐treated group. Hematoxylin and eosin (H&E) staining of injection sites also confirmed the presence of some inflammatory cells in the BG/OVA@EcN‐treated group (Figure 3j). Here, we noted that the supernatants from BG/OVA@EcN‐trained macrophages efficiently recruited DCs (Figure S10, Supporting Information). The enhanced numbers of monocytes/macrophages and DCs in the BG/OVA@EcN‐treated group might be due to their recruitment to the injection sites by the proinflammatory cytokines secreted by BG/OVA@EcN‐trained macrophages.

### 2.4 BG/OVA@EcN Efficiently Enhances Adaptive Antitumor Immunity

DCs, as one of the major APCs, are responsible for antigen phagocytosis, processing, and presentation to T cells, which is crucial for activating adaptive immune responses.^[^ ^26^ ^]^ Similar to macrophages, in vitro analysis also showed that BG/OVA@EcN exhibited the strongest phagocytosis by DC2.4 cells and murine bone marrow‐derived DCs (BMDCs) compared with OVA, BG + OVA, and OVA@EcN (Figure S11a–c, Supporting Information). The strong phagocytosis of BG/OVA@EcN resulted in the efficient antigen process and presentation of OVA by BMDCs, as evidenced by the highest percentages of H‐2K^b^‐SIINFEKL^+^ cells (Figure S12a,b, Supporting Information). Correspondingly, BG/OVA@EcN‐treated BMDCs exhibited the highest percentages of CD80^+^CD86^+^ (Figure S13a, Supporting Information) and MHCII^+^ cells (Figure S13b, Supporting Information), revealing the efficient DC maturation. Consistently, BG/OVA@EcN‐treated BMDCs showed the strongest capacity to activate CD8^+^ T cells, as indicated by the highest percentages of IFNγ^+^CD8^+^ T (Figure S13c, Supporting Information) and granzyme B (GzmB)^+^CD8^+^ T cells (Figure S13d, Supporting Information). These results indicated that BG/OVA@EcN efficiently induced DC mature and activated anti‐tumor T cell immunity.

**Figure 4:**
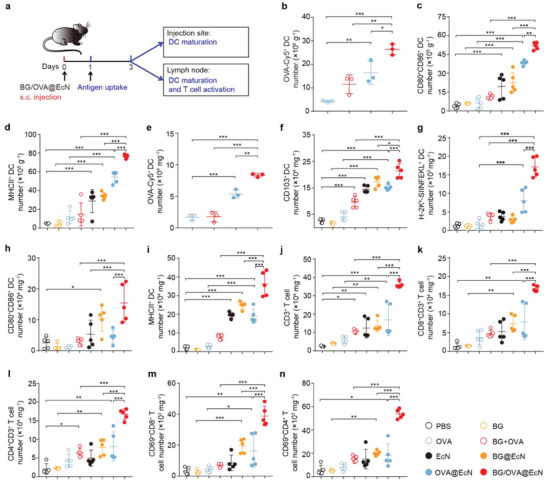
BG/OVA@EcN‐induced enhanced adaptive antitumor immunity. a) Schematic schedule of evaluating the in vivo immune responses triggered by BG/OVA@EcN. b) Numbers of Cy5^+^CD11c^+^ cells at the injection sites of C57BL/6 mice at 24 h after subcutaneous injection of OVA, BG + OVA, OVA@EcN, or BG/OVA@EcN (OVA was conjugated with Cy5) at the OVA dosage of 40 µg, BG dosage of 40 µg, and EcN dosage of 3 × 10^8^ CFU per mouse. Data are presented as mean ± SD (n = 3). c) Numbers of CD80^+^CD86^+^ DCs and d) MHCII^+^ DCs at the injection sites of C57BL/6 mice at 3 days after subcutaneous injection of PBS, BG, OVA, BG + OVA, EcN, BG@EcN, OVA@EcN, or BG/OVA@EcN at the OVA dosage of 40 µg, BG dosage of 40 µg, and EcN dosage of 3 × 10^8^ CFU per mouse. Data are presented as mean ± SD (*n* = 5). e) Numbers of Cy5^+^CD11c^+^ cells at the draining lymph nodes of C57BL/6 mice at 24 h after subcutaneous injection of OVA, BG + OVA, OVA@EcN, or BG/OVA@EcN (OVA was conjugated with Cy5) at the OVA dosage of 40 µg, BG dosage of 40 µg, and EcN dosage of 3 × 10^8^ CFU per mouse. Data are presented as mean ± SD (*n* = 3). f–n) Numbers of f) CD103^+^ DCs, g) H‐2K^b^‐SIINFEKL^+^ DCs, h) D80^+^CD86^+^ DCs, i) MHCII^+^ DCs, j) CD3^+^ T, k) CD8^+^ T, l) CD4^+^ T, m) the activated CD69^+^CD8^+^ T, and n) CD69^+^CD4^+^ T cells in draining lymph nodes of C57BL/6 mice at 3 days after treatment indicated in (a). Data are presented as mean ± SD (*n* = 5). *p* values are calculated using one‐way ANOVA followed by Tukey's HSD post‐hoc test. * *p* < 0.05, ** *p* < 0.01, *** *p* < 0.001.

To determine the phagocytosis of BG/OVA@EcN by DCs at the injection sites, C57BL/6 mice were subcutaneously injected with Cy5‐conjugated OVA, BG + OVA, OVA@EcN, or BG/OVA@EcN (**Figure** **4**a). Flow cytometric analysis showed that DCs at the injection sites of the BG/OVA@EcN‐treated group phagocytosed the most OVA antigen (Figure 4b), which might be owing to the highest number of DCs at the BG/OVA@EcN injection sites (Figure 3i) or BG/OVA@EcN‐promoted phagocytosis by DCs. Expectedly, the highest numbers of mature DCs (CD80^+^CD86^+^CD11c^+^CD45^+^ and MHCII^+^CD11c^+^CD45^+^ cells) at the injection sites were detected in BG/OVA@EcN‐treated group when the C57BL/6 mice were subcutaneously injected with PBS, BG, OVA, BG + OVA, EcN, BG@EcN, OVA@EcN, or BG/OVA@EcN (Figure 4c,d). DCs can be migrated from the injection sites to draining lymph nodes, allowing DCs to activate adaptive immunity. Consistently, the highest ratios of OVA^+^ DCs were detected in the lymph nodes of the BG/OVA@EcN‐treated group (Figure 4e). Meanwhile, BG/OVA@EcN treatment resulted in the highest numbers of migratory DCs (CD103^+^CD11c^+^CD45^+^ cells), H‐2K^b^‐SIINFEKL^+^ DCs, and mature DCs (CD80^+^CD86^+^CD11c^+^CD45^+^ and MHCII^+^CD11c^+^CD45^+^ cells) in the draining lymph nodes compared with PBS‐, BG‐, OVA‐, BG + OVA‐, EcN‐, BG@EcN‐, and OVA@EcN‐treated groups (Figure 4f–i). In addition, the highest numbers of CD3^+^ T (Figure 4j), CD8^+^ T (Figure 4k), CD4^+^ T (Figure 4l), activated CD69^+^CD8^+^ T (Figure 4m), and CD69^+^CD4^+^ T cells (Figure 4n) were found in the lymph nodes of BG/OVA@EcN‐treated group. Taken together, these results indicated that BG/OVA@EcN could efficiently promote adaptive antitumor immunity, which might be because BG/OVA@EcN induced trained immunity in macrophages to enhance DC recruitment and phagocytosis at the injection sites and the subsequent CD8^+^ T cell activation in lymph nodes.

### 2.5 BG/OVA@EcN Efficiently Induces Trained Immunity in Circulating Monocytes/Macrophages

**Figure 5:**
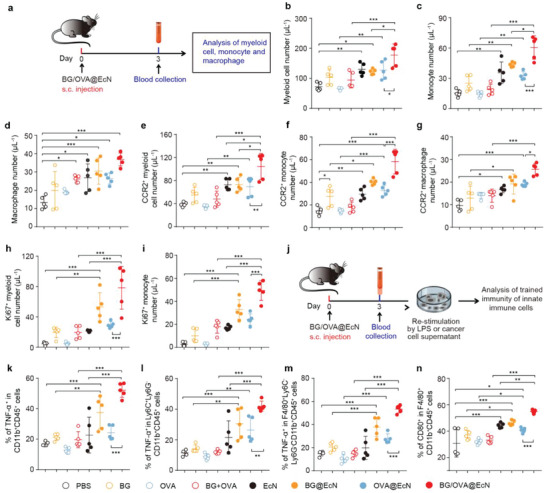
BG/OVA@EcN‐mediated trained immunity of circulating monocytes/macrophages in blood. a) Schematic schedule of evaluating the circulating myeloid cells, monocytes, and macrophages after BG/OVA@EcN treatment. b–i) Numbers of b) myeloid cells, c) monocytes, d) macrophages, e) CCR2^+^ myeloid cells, f) CCR2^+^ monocytes, g) CCR2^+^ macrophages, h) Ki67^+^ myeloid cells, and i) Ki67^+^ monocytes in the blood of C57BL/6 mice at 3 days after subcutaneous injection of PBS, BG, OVA, BG + OVA, EcN, BG@EcN, OVA@EcN, or BG/OVA@EcN at the OVA dosage of 40 µg, BG dosage of 40 µg, and EcN dosage of 3 × 10^8^ CFU per mouse. Data are presented as mean ± SD (*n* = 5). j) Schematic schedule of evaluating the ex vivo trained immune responses triggered by BG/OVA@EcN. k) Percentages of TNF‐α^+^ myeloid cells, l) TNF‐α^+^ monocytes, and m) TNF‐α^+^ macrophages after the blood cells from the above‐treated C57BL/6 mice on day 3 were re‐stimulated with 100 ng mL^−1^ LPS for 24 h. Data are presented as mean ± SD (*n* = 5). n) Percentages of CD80^+^ macrophages after the blood cells from the above‐treated C57BL/6 mice on day 3 were re‐stimulated with the supernatants of B16‐OVA cells for 24 h. Data are presented as mean ± SD (*n* = 5). *p* values are calculated using one‐way ANOVA followed by Tukey's HSD post‐hoc test. * *p* < 0.05, ** *p* < 0.01, *** *p* < 0.001.

It has been reported that BG can train circulating monocytes through reprogramming hematopoietic stem and progenitor cells, and the subsequently released cytokines, such as GM‐CSF and IL‐1β, are critical for BG‐induced myelopoiesis, which efficiently promoted antitumor immunity.^[^ ^9b^ ^]^ To determine the effects of BG/OVA@EcN on circulating monocytes/macrophages, C57BL/6 mice were subcutaneously injected with PBS, BG, OVA, BG + OVA, EcN, BG@EcN, OVA@EcN, or BG/OVA@EcN, and the numbers of immune cells in peripheral blood were determined by flow cytometry (**Figure** **5**a). Consistently, BG/OVA@EcN treatment led to the highest numbers of myeloid cells (Figure 5b), monocytes (Figure 5c), and macrophages (Figure 5d) compared with other groups, suggesting that BG/OVA@EcN efficiently increased the numbers of monocytes/macrophages in peripheral blood. Meanwhile, the numbers of migratory myeloid cells (Figure 5e), migratory monocytes (Figure 5f), and migratory macrophages (Figure 5g) in peripheral blood were the greatest in the BG/OVA@EcN‐treated group. In addition, the largest numbers of proliferating myeloid cells (Figure 5h) and monocytes (Figure 5i) expressing Ki67, a cell‐cycle‐related nuclear protein used as a proliferation marker, in peripheral blood were detected in BG/OVA@EcN‐treated group.

To determine whether BG/OVA@EcN mediated the trained immunity of circulating monocytes/macrophages, the same volume of blood was harvested from the mice subcutaneously injected with PBS, BG, OVA, BG + OVA, EcN, BG@EcN, OVA@EcN, or BG/OVA@EcN, and then re‐stimulated with LPS. Expectedly, the numbers of myeloid cells (Figure 5k), monocytes (Figure 5l), and macrophages expressing TNF‐α (Figure 5m) were significantly enhanced in EcN‐, BG@EcN‐, and OVA@EcN‐treated groups compared with PBS group. However, the highest numbers of myeloid cells (Figure 5k), monocytes (Figure 5l), and macrophages expressing TNF‐α (Figure 5m) were detected in BG/OVA@EcN‐treated group, indicating that BG/OVA@EcN efficiently enhanced trained monocytes/macrophages in peripheral blood. Tumor cells can secrete damage‐associated molecular patterns (DAMPs) or proinflammatory factors, functioning as the second stimulus in trained immunity.^[^ ^9a^ ^]^ The peripheral blood cells of mice after treatment as above were re‐stimulated with the supernatants of B16‐OVA cells. Consistently, BG/OVA@EcN‐treated group exhibited the largest numbers of macrophages expressing CD80 (Figure 5n), further confirming that BG/OVA@EcN efficiently promoted trained monocytes/macrophages in peripheral blood. Meanwhile, the enhanced CD80^+^ macrophages after tumor cell supernatant re‐stimulation implied that the systemic circulating monocytes/macrophages in the BG/OVA@EcN‐treated group might more likely differentiate into pro‐inflammatory macrophages after infiltration into the tumor tissues.

### 2.6 BG/OVA@EcN Exhibits Potent Anticancer Activity and Improved Tumor Microenvironment In Vivo

**Figure 6:**
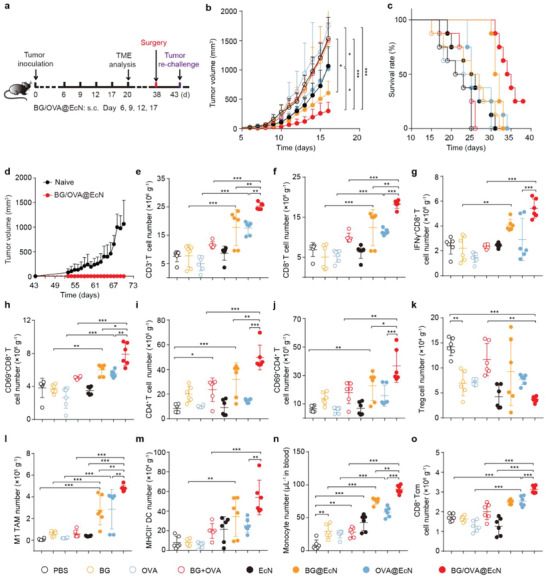
BG/OVA@EcN‐induced enhanced anticancer activity and antitumor immune response in subcutaneous B16‐OVA tumor‐bearing mice. a) Schematic schedule for anticancer experiments in subcutaneous B16‐OVA tumor‐bearing mice. b) Tumor growth curves of B16‐OVA tumor‐bearing mice after subcutaneous injection of PBS, BG, OVA, BG + OVA, EcN, BG@EcN, OVA@EcN, or BG/OVA@EcN at the OVA dosage of 40 µg, BG dosage of 40 µg, and EcN dosage of 3 × 10^8^ CFU per mouse as indicated in (a). Data are presented as mean ± SD (*n* = 6). c) Survival plots of B16‐OVA tumor‐bearing mice after treatment indicated in (a). (*n* = 8). d) Tumor growth curves after re‐challenge with B16‐OVA cells (6 × 10^5^ cells per mouse) in naïve mice or surviving BG/OVA@EcN‐treated mice undergoing removal of residual tumors indicated in (a). Data are presented as mean ± SD (*n* = 2 for BG/OVA@EcN‐treated mice, *n* = 3 for naïve mice). e–m) Numbers of e) CD3^+^ T cells, f) CD8^+^ T cells, g) IFN‐γ^+^CD8^+^ T cells, h) CD69^+^CD8^+^ T cells, i) CD4^+^ T cells, j) CD69^+^CD4^+^ T cells, k) Tregs, l) CD80^+^ TAMs, and m) MHCII^+^ DCs in tumor tissues of B16‐OVA tumor‐bearing mice after treatment indicated in (a). Data are presented as mean ± SD (*n* = 6). n) Numbers of monocytes in the blood of B16‐OVA tumor‐bearing mice after treatment indicated in (a). Data are presented as mean ± SD (*n* = 6). o) Numbers of CD8^+^ Tcm cells in spleens of B16‐OVA tumor‐bearing mice after treatment indicated in (a). Data are presented as mean ± SD (*n* = 6). *p* values are calculated using one‐way ANOVA followed by Tukey's HSD post‐hoc test. * *p* < 0.05, ** *p* < 0.01, *** *p* < 0.001.

To assess the immune protection effects induced by BG/OVA@EcN, C57BL/6 mice were subcutaneously injected with PBS, BG, OVA, BG + OVA, EcN, BG@EcN, OVA@EcN, or BG/OVA@EcN every 3 days for four times. The immunized mice were then challenged with B16‐OVA cells and the tumor growth was monitored (Figure S14a,b, Supporting Information). As expected, BG, OVA, BG + OVA, and EcN had no significant protective efficacy against tumor cells. BG@EcN and OVA@EcN showed tumor growth inhibition, but BG/OVA@EcN exhibited the strongest inhibitory effects and longest survival time, with 1 out of 6 mice being tumor‐free for over 60 days (Figure S14c, Supporting Information). Importantly, when this tumor‐free mouse in BG/OVA@EcN‐vaccinated group was re‐challenged with B16‐OVA cells on day 60, no visible secondary tumor growth was observed although continuous tumor growth was detected in naïve mice inoculated with the same number of B16‐OVA cells (Figure S14d, Supporting Information), suggesting that BG/OVA@EcN can exert good prophylactic therapy by generating antitumor immunity. Furthermore, the therapeutic efficacy of BG/OVA@EcN was evaluated in B16‐OVA tumor‐bearing mice (**Figure** **6**a). Consistently, BG/OVA@EcN showed the strongest anticancer activity, with 80.4% tumor inhibition compared with the PBS group (Figure 6b and Figure S15a–h, Supporting Information). Kaplan–Meier survival analysis showed that 25% of mice were still alive in BG/OVA@EcN‐treated group after 40 days when all mice in other groups died (Figure 6c), revealing that BG/OVA@EcN exhibited a promising potential as the therapeutic cancer vaccine. Meanwhile, 100% of the mice rejected the tumor re‐challenge up to 70 days when the surviving BG/OVA@EcN‐treated mice were re‐challenged with B16‐OVA cells after removing the residual tumors (Figure 6d). These results further revealed that BG/OVA@EcN had good antitumor activity by eliciting antitumor immune memory. No significant toxicity was determined in the BG/OVA@EcN‐treated group by body weight change (Figure S16, Supporting Information), H&E staining of major organs (Figure S17, Supporting Information), and serological analysis (Figure S18, Supporting Information).

To further investigate the BG/OVA@EcN‐induced antitumor immunity, the immune cells in draining lymph nodes and tumor tissues were investigated in B16‐OVA tumor‐bearing mice after subcutaneous injection of PBS, BG, OVA, BG + OVA, EcN, BG@EcN, OVA@EcN, or BG/OVA@EcN. Consistently, BG/OVA@EcN markedly promoted the numbers of mature CD80^+^CD86^+^ DCs (Figure S19a, Supporting Information), MHCII^+^ DCs (Figure S19b, Supporting Information), and H‐2K^b^‐SIINFEKL^+^ DCs (Figure S19c, Supporting Information), the activated CD69^+^CD8^+^ T (Figure S19d, Supporting Information) and CD69^+^CD4^+^ T cells (Figure S19e, Supporting Information), as well as the proliferating Ki69^+^CD8^+^ T cells (Figure S19f, Supporting Information) in the draining lymph nodes compared with BG@EcN‐ and OVA@EcN‐treated groups. Correspondingly, the numbers of CD3^+^ T (Figure 6e), CD8^+^ T (Figure 6f), and CD4^+^ T cells (Figure 6i) in tumor tissues were the highest in the BG/OVA@EcN‐treated group. Moreover, BG/OVA@EcN exhibited the strongest ability to promote tumor infiltration of the activated IFN‐γ^+^CD8^+^ T (Figure 6g) and CD69^+^CD8^+^ T cells (Figure 6h), as well as the activated CD69^+^CD4^+^ T cells (Figure 6j), while decreasing the numbers of Tregs (Foxp3^+^CD25^+^CD4^+^CD3^+^CD45^+^ cells, Figure 6k). In addition, BG/OVA@EcN significantly increased the numbers of M1‐like TAMs (Figure 6l) and mature DCs (Figure 6m) in the tumor tissues, which might be due to the fact that the enhanced trained monocytes (Figure 6n) in the peripheral blood were infiltrated into the tumor tissues and differentiated into pro‐inflammatory M1‐like TAM or DCs. These data indicated that BG/OVA@EcN efficiently enhanced the antitumor immunity and improved tumor immunosuppressive microenvironment. Besides, BG/OVA@EcN significantly enhanced the numbers of CD8^+^ central memory T (Tcm) cells (CD44^+^CD62L^+^CD8^+^CD3^+^CD45^+^ T cells) in the spleens (Figure 6o), confirming that BG/OVA@EcN efficiently induced immunological memory.

### 2.7 Personalized BG/Ag@EcN Generates Strong Antitumor Immune Memory and Inhibits Post‐Surgical Tumor Recurrence

**Figure 7:**
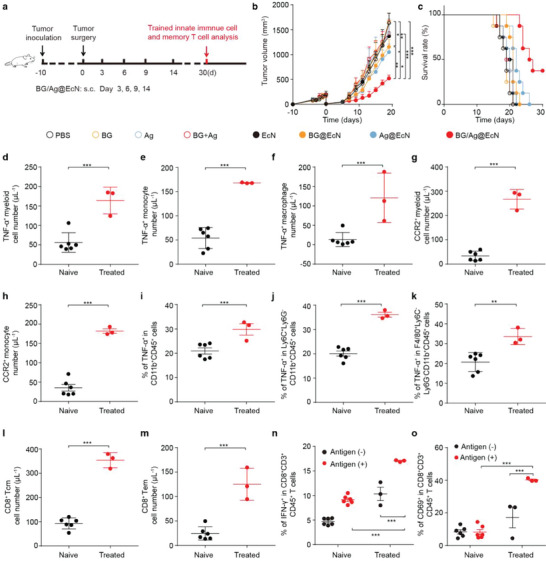
Personalized BG/Ag@EcN for inhibiting post‐surgical tumor recurrence in orthotopic 4T1 tumor‐bearing mice. a) Schematic schedule for antitumor recurrence experiments in orthotopic 4T1 tumor‐bearing mice. b) Tumor growth curves of 4T1 tumor‐bearing mice undergoing surgical tumor resection after subcutaneous injection of PBS, BG, Ag, BG + Ag, EcN, BG@EcN, Ag@EcN, or BG/Ag@EcN at the Ag dosage of 40 µg, BG dosage of 40 µg and EcN 3 × 10^8^ CFU per mouse as indicated in (a). Data are presented as mean ± SD (*n* = 6). *p*‐values are calculated using one‐way ANOVA followed by Tukey's HSD post‐hoc test. c) Survival plots of 4T1 tumor‐bearing mice undergoing surgical tumor resection after treatment indicated in (a). (*n* = 8). d–h) Numbers of d) TNF‐α^+^ myeloid cells, e) TNF‐α^+^ monocytes, f) TNF‐α^+^ macrophages, g) CCR2^+^ myeloid cells, and h) CCR2^+^ monocytes in the blood of naïve mice and survival 4T1 tumor‐bearing mice undergoing surgical tumor resection after treatment indicated in (a). Data are presented as mean ± SD (*n* = 3 for BG/Ag@EcN‐treated mice, *n* = 6 for naïve mice). *p*‐values are calculated using an unpaired two‐tailed Student's *t*‐test. i–k) Percentages of i) TNF‐α^+^ myeloid cells, j) TNF‐α^+^ monocytes, and k) TNF‐α^+^ macrophages after the blood cells of the naïve mice and survival 4T1 tumor‐bearing mice undergoing surgical tumor resection after treatment indicated in (a) were re‐stimulated with 100 ng mL^−1^ LPS for 24 h. Data are presented as mean ± SD (*n* = 3 for BG/Ag@EcN‐treated mice, *n* = 6 for naïve mice). *p*‐values are calculated using an unpaired two‐tailed Student's *t*‐test. l) Percentages of CD8^+^ Tcm cells and m) CD8^+^ Tem cells in the blood of naïve mice and survival 4T1 tumor‐bearing mice undergoing surgical tumor resection after treatment indicated in (a). Data are presented as mean ± SD (*n* = 3 for BG/Ag@EcN‐treated mice, *n* = 6 for naïve mice). *p*‐values are calculated using an unpaired two‐tailed Student's *t*‐test. n) Percentages of IFN‐γ^+^CD8^+^ T cells and o) CD69^+^CD8^+^ T cells after the blood cells of the naïve mice and survival 4T1 tumor‐bearing mice undergoing surgical tumor resection after treatment indicated in a was treated with or without 4T1 cell lysates for 72 h. Data are presented as mean ± SD (*n* = 3 for BG/Ag@EcN‐treated mice, *n* = 6 for naïve mice). *p*‐values are calculated using two‐way ANOVA with Bonferroni correction. * *p* < 0.05, * * *p* < 0.01, *** *p* < 0.001.

Surgical resection is the first‐line treatment option for clinical early‐stage solid tumors.^[^ ^27^ ^]^ However, residual tumor cells or circulating tumor cells (CTCs) may cause lethal tumor recurrence and metastasis.^[^ ^28^ ^]^ Activating tumor‐specific immune responses to prevent tumor relapse after surgery represents a promising strategy for cancer treatment.^[^ ^29^ ^]^ To determine the anti‐recurrence effects of personalized tumor vaccines after surgical tumor resection, 90% of tumors were removed from orthotopic 4T1 tumor‐bearing mice, and the tumor cell membranes as the source of tumor antigens (Ag) which were collected from the resected 4T1 tumors were simply mixed with PEI@EcN and BG to fabricate the BG/Ag@EcN. Confocal microscopic images and zeta potential of BG/Ag@EcN confirmed its successful construction (Figure S20a,b, Supporting Information). 4T1 tumor‐bearing mice after tumor resection were subcutaneously injected with PBS, BG, Ag, BG + Ag, EcN, BG@EcN, Ag@EcN or BG/Ag@EcN on days 3, 6, 9, and 14, and the tumor growth was determined (**Figure** **7**a,b and Figure S21a–h, Supporting Information). As expected, the residual tumors of mice treated with PBS, BG, Ag, BG + Ag, or EcN grew fast and exhibited similar tumor growth curves. Treatment with BG@EcN, Ag@EcN, or BG/Ag@EcN significantly inhibited tumor growth after surgical tumor resection. However, the strongest anticancer effects were detected in the BG/Ag@EcN‐treated group. Meanwhile, Kaplan–Meier survival analysis showed that 37.5% of mice were still alive in BG/Ag@EcN‐treated group after 30 days when all mice in other groups died (Figure 7c), suggesting that BG/Ag@EcN exhibited potent anti‐recurrence capacity after tumor resection. No obvious body weight loss was observed during BG/Ag@EcN treatment (Figure S22, Supporting Information), further confirming the good biosafety of BG/Ag@EcN.

To investigate BG/Ag@EcN‐induced trained immunity in the peripheral blood of 4T1 tumor‐bearing mice undergoing surgical tumor resection, the same volume of peripheral blood was collected from naïve mice and the surviving mice treated with BG/Ag@EcN. Consistently, the numbers of myeloid cells (Figure 7d), monocytes (Figure 7e), and macrophages expressing TNF‐α (Figure 7f) were significantly enhanced in the BG/Ag@EcN‐treated group compared with those in the naïve mice, suggesting that BG/Ag@EcN elicited greater pro‐inflammatory cytokine responses in myeloid cells, monocytes, and macrophages. Meanwhile, significantly higher numbers of migrating CCR2^+^ myeloid cells (Figure 7g) and monocytes (Figure 7h) were detected in the BG/Ag@EcN‐treated group than those in the naïve mice. Furthermore, when these collected peripheral blood was then re‐stimulated with LPS, the ratios of myeloid cells (Figure 7i), monocytes (Figure 7j), and macrophages expressing TNF‐α (Figure 7k) in the blood of BG/Ag@EcN‐treated group were obviously higher than those in naïve mice, confirming that BG/Ag@EcN efficiently induced trained immunity in monocytes/macrophages. In addition, BG/Ag@EcN treatment significantly enhanced the ratios of CD8^+^ Tcm cells (Figure 7l) and CD8^+^ effector memory T (Tem) cells (CD44^+^CD62L^‐^CD8^+^CD3^+^CD45^+^ T cells) (Figure 7m) in blood compared with those of naïve mice, revealing that BG/Ag@EcN might efficiently induce immunological memory in 4T1 tumor‐bearing mice undergoing surgical tumor resection. To further verify this, 4T1 cell lysates were used to stimulate CD8^+^ T cells from blood (Figure 7n,o) of the naïve mice and the surviving BG/Ag@EcN‐treated mice, and then the activation of CD8^+^ T cells was analyzed by flow cytometry. Consistently, re‐stimulation with 4T1 antigen significantly increased the ratios of IFN‐γ^+^CD8^+^ and CD69^+^CD8^+^ T cells of BG/Ag@EcN‐treated group compared with those of the naïve mice, confirming that BG/Ag@EcN treatment might generate long‐term immune memory to inhibit tumor recurrence after surgical tumor resection.

## 3 Discussion and Conclusion

Cancer vaccines hold great potential for inhibiting tumor growth and metastasis by manipulating the immune system to recognize and eliminate tumor cells.^[^ ^30^ ^]^ However, cancer vaccines have shown suboptimal clinical benefit in cancer patients due to insufficient antitumor immune response.^[^ ^31^ ^]^ Although several combination strategies have been used to improve the therapeutic efficacy of cancer vaccines, many of them focus only on T cell responses and cannot simultaneously mobilize the innate immune system.^[^ ^32^ ^]^ Recent works have shown that the activation of adaptive T cell responses and long‐term protective immune memory are tightly associated with innate immunity,^[^ ^32^, ^33^ ^]^ especially trained immunity which is characterized by “immune memory” with a more robust innate immune response initiated upon recognition of secondary homologous or heterologous stimulator.^[^ ^11^ ^]^ It is quite promising to develop cancer vaccines activating trained immunity to establish and maintain robust and long‐lasting antigen‐specific adaptive immunity.

Recently, bacteria have been used as the platform of cancer vaccines due to easy genetic engineering as well as functioning as immune adjuvants.^[^ ^17^, ^34^ ^]^ For example, bacteria can be specifically engineered to express antigens or adjuvants to activate antitumor immunity.^[^ ^18^, ^35^ ^]^ However, considering the potential risk of triggering mutations via horizontal gene transfer, using genetically engineered bacteria to treat cancer remains a major concern.^[^ ^36^ ^]^ The attachment of antigens or adjuvants onto the surface of bacteria offers an alternative to achieving ideal cancer vaccines. In this study, we developed a facile strategy to prepare EcN‐based cancer vaccine with trained immunity and adaptive immunity by engineering inactivated EcN with PEI to obtain positively charged EcN, followed by incubating with BG and tumor antigens for synergistic anticancer effects. EcN not only acted as a carrier to enhance the tumor antigen deposition at the injection sites but also functioned as an immune adjuvant and trained immunity inducer due to the presence of PAMP. BG, also a trained immunity inducer, triggered trained immunity in macrophages in combination with EcN at the injection sites. Our results showed that BG/OVA@EcN efficiently enhanced the accumulation and phagocytosis by macrophages at the injection sites, which accounts for its stronger antitumor immunity than single OVA, BG, EcN, or even probably a combination of OVA, BG, and EcN. This preparation method of EcN‐based cancer vaccines was simple and universal, loading not only model antigen OVA but also personalized tumor antigens to achieve strong antitumor immunity.

The adaptive antitumor immunity of cancer vaccines is restricted by several cascading events, including antigen phagocytosis by DCs, antigen presentation, and T cell activation.^[^ ^2d^ ^]^ Efficiently improving these events will contribute to enhancing the antitumor immunity of cancer vaccines. The trained innate immune cells can induce a significant increase in the levels of proinflammatory cytokines, directing the adaptive immunity toward a more effective response against tumors.^[^ ^7^, ^33^, ^37^ ^]^ For example, proinflammatory cytokines, such as TNF‐α, IL‐6, IL‐1β, CCL2, and CCL4 released from innate cells can recruit DCs and promote DC maturation and T cell activation.^[^ ^3^, ^13^ ^]^ In this work, BG/OVA@EcN efficiently induced trained immunity of macrophages, as evidenced by the enhanced expressions of proinflammatory cytokines TNF‐α after stimulating with secondary stimulus and increased phagocytosis of tumor cells in vitro, as well as the enhanced numbers of proinflammatory macrophages at the injection sites after subcutaneous injection. The proinflammatory cytokines released by BG/OVA@EcN‐trained macrophages might efficiently recruit monocytes/macrophages and DCs, resulting in enhanced numbers of migratory monocytes/macrophages and DCs at the injection sites. The recruited DCs efficiently phagocytosed BG/OVA@EcN to promote DC maturation and CD8^+^ T cell activation. The recruited monocytes/macrophages might be further trained by BG/OVA@EcN at the injection sites, generating more proinflammatory cytokines to strengthen this process. Thus, in vivo immune microenvironment analysis showed that subcutaneous injection of BG/OVA@EcN effectively promoted and activated CD8^+^ T cells in lymph nodes, achieving a collaborative innate‐adaptive immune response. Besides BG‐induced trained immunity in macrophages, the anticancer effects of BG‐induced trained immunity were reported to be associated with transcriptomic and epigenetic rewiring of granulopoiesis and neutrophil reprogramming toward an anti‐tumor phenotype.^[^ ^38^ ^]^ Our work found that no significant increase in the number of neutrophils was detected at the injection sites (Figure S23a, Supporting Information), blood (Figure S23b, Supporting Information), and tumor tissues (Figure S23c, Supporting Information) of BG/OVA@EcN‐treated group. However, when the neutrophils in blood were isolated and then stimulated with LPS, significantly increased ratio of TNF‐α^+^ neutrophils (TNF‐α^+^Ly6G^+^Ly6**c** ^‐^CD45^+^CD11b^+^ cells) were observed in BG/OVA@EcN‐treated group (Figure S23d, Supporting Information), indicating that BG/OVA@EcN might induce trained immunity of neutrophils. The detailed effects and mechanisms of the BG/OVA@EcN‐induced trained immunity in neutrophils need to be further investigated.

Antigen‐specific T cells must enter the tumor microenvironment from the draining lymph nodes to kill the antigen‐expressing cancer cells.^[^ ^2d^ ^]^ However, the immunosuppressive tumor microenvironment which was mainly established through the recruitment of immunosuppressive cells, including TAMs and Tregs, induced cytotoxic T cell exclusion and energy to render cancer resistant to immunotherapy.^[^ ^39^ ^]^ Thus, combining the reprogramming of tumor immunosuppressive microenvironment toward an immunoresponsive state with cancer vaccines exhibited the potential to improve cancer therapeutics. Recent works have shown that the induction of trained immunity of innate cells resulted in enhanced trained monocyte numbers that differentiated into antitumor macrophages, shifting tumor immunosuppressive microenvironment to a proinflammatory antitumor state.^[^ ^9^, ^15^ ^]^ Here, we found that BG/OVA@EcN not only efficiently increased the numbers of trained monocytes/macrophages in the peripheral blood of C57BL/6 mice, but also increased the numbers of migratory myeloid cells, monocytes, and macrophages as well as the numbers of proliferative myeloid cells and monocytes in peripheral blood. Meanwhile, the BG/Ag@EcN‐induced enhanced trained immunity in peripheral blood was further confirmed in 4T1 tumor‐bearing mice undergoing surgical tumor resection. Based on our data and the previous reports,^[^ ^9^, ^10^, ^15^ ^]^ the increase in the numbers of trained monocytes/macrophages in peripheral blood might be due to the migration of the released BG‐induced trained bone marrow hematopoietic stem cells and progenitor cells, or the released BG‐induced direct training of monocytes/macrophages in peripheral blood, which remained to be further elucidated. The circulating monocytes/macrophages bearing a trained immunity phenotype might traffic into the tumors to phagocytose tumor cells. Meanwhile, the trained monocytes/macrophages at the tumor tissues might differentiate into the proinflammatory M1 macrophages to remodel the tumor immunosuppressive microenvironment, as evidenced by the fact that the re‐stimulation of BG/OVA@EcN‐trained peripheral blood cells with the supernatants of B16‐OVA cells generated more CD80^+^ macrophages. Meanwhile, the increased numbers of M1‐like TAMs after BG/OVA@EcN treatment in B16‐OVA tumor‐bearing mice further confirmed BG/OVA@EcN‐improved tumor immunosuppressive microenvironment.

The findings of our research have underscored the pivotal role played by BG/Ag@EcN‐induced trained immunity in enhancing the immune response against different tumors by integrating tumor‐specific antigens. Although the successful clinical translation of BG/Ag@EcN‐induced trained immunity holds great promise for improving the immunotherapeutic approaches in cancer treatment, several crucial considerations come into play. First and foremost, the choice of the appropriate dosage of BG/Ag@EcN is of paramount importance. Striking the right balance between an effective immune response and avoiding potential side effects will be essential in clinical trials. Secondly, it will be crucial to establish the safety and efficacy of this system in human subjects, although it has been tested with promising results in animal models. Rigorous clinical trials will need to be conducted to determine the optimal dosing regimen and to assess the overall impact on patients' antitumor immunity. Moreover, the use of EcN, which is a non‐pathogenic strain of *E. coli* (Nissle 1917), should also be carefully evaluated for its safety and tolerability in a clinical context.

In summary, the data in this study show that BG/OVA@EcN efficiently trains macrophages at the subcutaneous injection sites to recruit monocytes/macrophages and DCs, as well as promote the subsequent DC phagocytosis and antigen presentation for enhanced adaptive antitumor immunity. Meanwhile, BG/OVA@EcN trains circulating monocytes/macrophages for reprogramming tumor immunosuppressive microenvironment. BG/OVA@EcN achieves remarkable prophylactic and therapeutic efficacy to suppress established tumor growth by eliciting strong T cell‐mediated antitumor immune responses and long‐term immune memory. Moreover, EcN is used to deliver BG and autologous tumor antigens to inhibit postoperative tumor recurrence by inducing trained innate immunity and immune memory. Our work demonstrates a strong and personalized cancer vaccine with significant clinical translation potential for enhanced anticancer treatment.

## 4 Experimental Section

### Materials

PEI with molecular weights of 25 kDa, fetal bovine serum (FBS) and collagenase type I were purchased from Gibco Life Technologies (New York, USA). N‐(3‐Dimethylaminopropyl)‐N’‐ethylcarbodiimide hydrochloride (EDC), and N‐Hydroxysuccinimide (NHS) were purchased from Aladdin (Shanghai, China). *T. versicolor*‐derived BG was purchased from InvivoGen (Toulouse, France), consisting of a highly ramified glucan portion containing a beta 1–4 main chain and beta 1–3 side chain, with beta 1–6 side chains covalently linked to a polypeptide portion rich in aspartic, glutamic, and other amino acids. OVA and FTIC‐OVA were obtained from Sigma‐Aldrich (St Louis, USA) and Solarbio (Beijing, China), respectively. Dulbecco's modified Eagle's medium (DMEM), Roswell Park Memorial Institute (RPMI) 1640 medium, PBS, and penicillin‐streptomycin were purchased from HyClone (Logan, USA). Mouse granulocyte‐macrophage colony‐stimulating factor (GM‐CSF), IL‐4, and macrophage colony‐stimulating factor (M‐CSF) and antibodies used for flow cytometric analysis were purchased from BioLegend (San Diego, USA). Protease inhibitor cocktail tablets were purchased from Roche (Basel, Switzerland).

### Cell Culture and Animals

B16‐OVA cells were kindly provided by Dr. Bo Huang (Institute of Basic Medical Sciences, Chinese Academy of Medical Sciences, Beijing, China). RAW264.7, B16F10, THP‐1, HUVECs, NIH/3T3, and 4T1 cells were purchased from the Chinese Academy of Sciences (Shanghai, China). DC2.4 cells were purchased from the Fenghui Biotechnology Co., Ltd. (Hunan, China). RAW264.7, DC2.4, HUVECs, and NIH/3T3 cells were cultured in DMEM medium, and B16‐OVA, B16F10, THP‐1, and 4T1 cells were cultured in RPMI 1640 medium supplemented with 10% FBS and 1% penicillin‐streptomycin at 37 °C under 5% CO_2_. BMDMs and BMDCs were generated from the 6‐week‐old C57BL/6 female mice as described. Briefly, mouse bone marrow cells were flushed from the tibia and femur with PBS containing 2% FBS, treated with red cell lysis solution (Biosharp, Hefei, China), and then cultured in complete RPMI 1640 medium containing 30 ng mL^−1^ recombinant mouse M‐CSF or 10 ng mL^−1^ recombinant mouse GM‐CSF plus 5 ng mL^−1^ IL‐4 for 5 days to acquire the macrophages or DCs, respectively. Female BALB/c mice and C57BL/6 mice (5–6 weeks of age) were purchased from Beijing Vital River Laboratory Animal Technology Co., Ltd. (Beijing, China). All animal experiments were approved by the Institutional Animal Care and Use Committee at Tongji Medical College, Huazhong University of Science and Technology (Wuhan, China) (approval IACUC Number: 3428), and complied with the recommendations of the academy's animal research guidelines.

### Preparation and Characterization of BG/OVA@EcN

EcN was kindly provided by Prof. Zhi Liu (Huazhong University of Science and Technology, Wuhan, China). EcN was aerobically grown and maintained in Luria–Bertani (LB) medium (10 g l^−1^ tryptone, 5 g l^−1^ yeast extract, and 10 g l^−1^ NaCl) at 37 °C. When the bacterial OD600 reached about 1.0,^[^ ^40^ ^]^ about 3 × 10^8^ CFU of EcN were dispersed in 1 mL of PBS at 100 °C for 10 min to obtain the inactivated bacteria. The bacterial viability was evaluated by DAPI and PI double staining assay. Briefly, EcN and inactivated EcN were incubated with a sufficient volume of DAPI (0.5 µg mL^−1^) and PI (1.5 µmol L^−1^) working solution at room temperature in the dark for 30 min. Then, the samples were washed twice with PBS and observed on an FV3000 confocal laser scanning microscope (CLSM) (Olympus, Japan).

The inactivated EcN was dispersed in PBS containing 3 mg mL^−1^ PEI, 25 mg mL^−1^ EDC, and 20 mg mL^−1^ NHS. The resulting mixtures were shaken at 37 °C for 30 min, washed with PBS three times, and then centrifuged at 6000 g for 3 min. 40 µg OVA and 40 µg BG dispersed in 200 µL PBS were then added to the pellets, shaken at 37 °C for 30 min, and then centrifuged at 6000 g for 3 min to obtain EcN loading OVA and BG (BG/OVA@EcN). EcN loading BG or OVA (BG@EcN or OVA@EcN) was obtained according to the same protocol, except that only 40 µg OVA or 40 µg BG was added to the EcN pellets. The loading efficiency of OVA or BG was determined from the difference in mass between the initial incubation solutions and the reserved supernatants. The remaining amounts of OVA and BG in the supernatants were determined by using a bicinchoninic acid (BCA) kit (Beyotime, Shanghai, China) and Red staining of Congo (Solarbio, Beijing, China), respectively. The morphology of BG/OVA@EcN was observed by an HT7700 TEM (Hitachi, Japan). The zeta potential of BG/OVA@EcN was detected by using a Zetasizer Nano ZS90 (Malvern, UK).

### Cytotoxicity Assessment

The cytotoxicity of BG/OVA@EcN against DC2.4, RAW264.7, NIH/3T3, and HUVECs cells was determined by CCK‐8 assay (Dojindo, Kumamoto, Kyushu, Japan). Typically, the cells were seeded in a 96‐well plate and incubated overnight at 37 °C. BG/OVA@EcN at different concentrations were added to each well and incubated in 100 µL RPMI 1640 medium at 37 °C for 12 h. After the cells were washed with PBS three times, 10 µL CCK‐8 was added to each well and further incubated at 37 °C for 4 h. The absorbance at 450 nm was measured on a Multiskan FC microplate reader (Thermo Scientific, Waltham, USA).

### Antigen Uptake by RAW264.7 and DC2.4 Cells

RAW264.7 and DC2.4 cells were treated with OVA, BG + OVA, OVA@EcN, or BG/OVA@EcN (OVA was conjugated with FITC) at the concentration of 3 × 10^7^ CFU mL^−1^ EcN, 4 µg mL^−1^ OVA, and 4 µg mL^−1^ BG for 4 h. The cells were washed with PBS three times and the intracellular FITC fluorescence was detected by CytoFLEX S flow cytometry (Beckman Coulter, USA). For confocal microscopic analysis, the treated cells were fixed with 4% formaldehyde for 10 min, permeabilized with 0.5% Triton X‐100 for 5 min, and incubated with 100 nM TRITC‐labeled phalloidin at room temperature for 30 min in the dark. The cells were washed with PBS three times and observed by an FV3000 CLSM.

### Maturation of BMDMs and BMDCs

BMDMs and BMDCs were treated with PBS, BG, OVA, BG + OVA, EcN, BG@EcN, OVA@EcN, or BG/OVA@EcN at the concentration of 3 × 10^7^ CFU mL^−1^ EcN, 4 µg mL^−1^ OVA, and 4 µg mL^−1^ BG for 12 h. For determination of macrophage maturation, BMDMs were then collected and stained with anti‐F4/80‐PE/Cy7 (Biolegend, cat. No 123114, clone BM8), anti‐CD80‐PE (Biolegend, cat. No 104708, clone 16‐10A1) and anti‐CD86‐APC (Biolegend, cat. No 105012, clone GL‐1) antibodies. For determination of BMDC maturation, BMDCs were collected and stained with anti‐CD11c‐FITC (Biolegend, cat. No 117306, clone N418), anti‐CD80‐PE, anti‐CD86‐APC, anti‐MHCII‐Percp/Cyanine5.5 (Biolegend, cat. No 107626, clone M5/114.15.2) or anti‐H‐2K^b^‐SIINFEKL‐PE antibodies (Biolegend, cat. No 141604, clone 25‐D1.16). All antibodies were diluted according to the instructions and incubated with the cells at room temperature for 30 min. The cells were washed with PBS and then analyzed by CytoFLEX flow cytometry.

### In Vitro T Cell Activation

CD8^+^ T cells were isolated from the spleens of 6‐week‐old healthy C57BL/6 female mice using the CD8^+^ T cell MACS negative selection kit (Biolegend, San Diego, USA) according to the manufacturer's instructions, and then cultured in complete RPMI 1640 medium supplemented with 20 ng mL^−1^ IL‐2 for 6 days. The CD8^+^ T cells were co‐cultured with the above mature BMDCs at the DC/T cell ratio of 1:10 for another 24 h. The cells were stimulated with Cell Activation Cocktail (with Brefeldin A, Biolegend, No 423303) for 2 h. The cells were stained with anti‐mouse CD8‐PE/Cyanine 7 antibody (Biolegend, No 100722, clone 53–6.7), and then fixed with intracellular staining fixation buffer (Biolegend, No 420801) and permeabilized with permeabilization wash buffer (Biolegend, No 421002) according to the manufacturer's instructions. The cells were further stained with anti‐mouse Granzyme‐B‐Alexa Fluor 647 (Biolegend, No 515406, clone GB11) or anti‐IFN‐γ‐PE (Biolegend, cat. No 505808, clone XMG1.2) for 30 min. The cells were washed with PBS and subjected to flow cytometric analysis.

### Real‐Time RT‐PCR

BMDMs were treated with PBS, BG, OVA, BG + OVA, EcN, BG@EcN, OVA@EcN, or BG/OVA@EcN at the concentration of 3 × 10^7^ CFU mL^−1^ EcN, 4 µg mL^−1^ OVA, and 4 µg mL^−1^ BG for 12 h. The cells were washed with PBS and collected for the mRNA expression analysis of macrophage activation genes (IL‐1β, TNF‐α, IL‐6, and IL‐12 by real‐time RT‐PCR. The used primer sequences for RT‐PCR are as follows: Mouse GAPDH (F: 5′‐GTTCCTACCCCCAATGTGTCC‐3′, R: 5′‐TAGCCCAAGATGCCCTTCAGT‐3′); Mouse IL‐1β (F: 5′‐GCAACTGTTCCT GAACTCAACT‐3′, R: 5′‐ATCTTTTGGGGTCCGTCAACT‐3′); Mouse TNF‐α (F: 5′‐CCCTCACACTCAGATCATCTTCT‐3′, R: 5′‐GCTACGACGTGGGCTACAG ‐3′); Mouse IL‐6 (F: 5′‐TAGTCCTTCCTACCCCAATTTCC‐3′, R: 5′‐TTGGTCCTTA GCCACTCCTTC‐3′); Mouse IL‐12 (F: 5′‐TGCCTTGGTAGCATCTATGAGG‐3′, R: 5′‐CGCAGAGTCTCGCCATTATGAT‐3′) and synthesized by Tsingke Biotechnology Co., Ltd (Beijing, China).

### Macrophage Training by BG/OVA@EcN

In vitro macrophage training was performed according to the well‐established model.^[^ ^41^ ^]^ Briefly, BMDMs were obtained by isolating the bone marrow cells from healthy C57BL/6 mice and then cultured in RPMI 1640 medium supplemented with 10% FBS and 30 ng mL^−1^ M‐CSF for 3 days. BMDMs and THP‐1 cells and then treated with PBS, BG, OVA, BG + OVA, EcN, BG@EcN, OVA@EcN, or BG/OVA@EcN at the concentration of 3 × 10^7^ CFU mL^−1^ EcN, ^4^ µg mL^−1^ OVA, and 4 µg mL^−1^ BG for 12 h. After the first training, the cells were washed with PBS and incubated with fresh RPMI 1640 medium containing 30 ng mL^−1^ M‐CSF. The cells were allowed to rest for the following 5 days. On day 8, the cells were re‐stimulated with 100 ng mL^‐1^ LPS for 24 h and then stimulated with Cell Activation Cocktail (Biolegend, cat. No 423304) for 2 h. For determination of BMDM training, BMDMs were washed with PBS and then stained with anti‐CD11b‐FITC (Biolegend, cat. No 101206, clone M1/70), anti‐F4/80‐PE/Cy7, and anti‐CD80‐PE, followed by fixing with intracellular staining fixation buffer and permeabilizing with permeabilization wash buffer according to the manufacturer's instructions. The cells were further stained with anti‐TNF‐α‐Brilliant Violet 421 antibody (Biolegend, cat. No 506328, clone MP6‐XT22) for 30 min. For determination of THP‐1 training, the cells were washed with PBS and then stained with anti‐human CD11b‐FITC (Biolegend, cat. No 301329, clone ICRF44) and anti‐human CD14‐APC (Biolegend, cat. No 367118, clone 63D3), followed by fixing and permeabilizing with intracellular staining fixation buffer and permeabilization wash buffer, respectively. The cells were further stained with anti‐human TNF‐α‐PE antibody (Biolegend, cat. No 376204, clone W19063E) for 30 min. The cells were harvested for flow cytometric analysis.

### Phagocytosis of Tumor Cells by Trained Macrophages

BMDMs were placed on sterile glass coverslips for attachment. BMDMs were treated with PBS, BG, OVA, BG + OVA, EcN, BG@EcN, OVA@EcN, or BG/OVA@EcN at the concentration of 3 × 10^7^ CFU mL^‐1^ EcN, 4 µg mL^−1^ OVA, and 4 µg mL^−1^ BG for 12 h. After resting for 5 days, the above‐trained BMDMs were labeled with 1 µM CFSE (Thermo Fisher Scientific, Waltham, MA, USA) and then co‐cultured with 1 µM DiR (Yeasen, Shanghai, China)‐labeled B16‐OVA or B16F10 cells at the ratio of 1:1 for 4 h. The phagocytosis of tumor cells by the trained macrophages was calculated as the ratio of CFSE^+^DiR^+^ cells among CFSE^+^ BMDMs by flow cytometry and observed by an FV3000 CLSM.

### In Vitro DC Recruitment

DC recruitment was performed using a 5‐µm transwell filter (Corning Costar, Acton, MA, USA). BMDMs were treated with PBS, BG, OVA, BG + OVA, EcN, BG@EcN, OVA@EcN, or BG/OVA@EcN at the concentration of 3 × 10^7^ CFU mL^−1^ EcN, ^4^ µg mL^−1^ OVA, and 4 µg mL^−1^ BG for 12 h, and the supernatants were collected as the conditional media and added to the bottom chambers. BMDCs (1 × 10^5^ cells) were seeded in the top chambers. After 8 h incubation, the CD11c^+^ cells from the bottom chambers were detected by flow cytometry.

### Antigen Accumulation at the Injection Sites

Healthy C57BL/6 mice were subcutaneously injected with Cy5‐labeled OVA, BG + OVA, OVA@EcN, or BG/OVA@EcN at the OVA dosage of 40 µg, BG dosage of 40 µg, and EcN dosage of 3 × 10^8^ CFU per mouse. At different time intervals, the mice were observed using an IVIS Lumina XR system (Caliper Life Sciences, USA). At 24 h after injection, the mice were euthanized, and the tissues at the injection sites were extracted, processed through mechanical disruption, and incubated with collagenase I (0.8 mg mL^−1^) and recombinant DNase I (100 U mL^−1^) in RPMI 1640 medium at 37 °C for 30 min, followed by filtering through a nylon 200‐mesh filters to obtain the single cell suspensions. The cells were stained with anti‐CD11b‐Percp/Cy5.5 (Biolegend, cat. No 101228, clone M1‐70), anti‐F4/80‐PE/Cy7 antibody, or anti‐CD11c‐FITC antibody. The Cy5 fluorescence in CD11b^+^F4/80^+^ cells or CD11c^+^ cells was determined by flow cytometry.

### Flow Cytometric Assay for Immune Response

Healthy C57BL/6 mice or tumor‐bearing mice were subcutaneously injected with PBS, BG, OVA, BG + OVA, EcN, BG@EcN, OVA@EcN, or BG/OVA@EcN at the OVA dosage of 40 µg, BG dosage of 40 µg, and EcN dosage of 3 × 10^8^ CFU per mouse. At the indicated times, the injection sites or tumor tissues were cut into small pieces and then incubated with collagenase I (0.8 mg mL^−1^) and recombinant DNase I (100 U mL^−1^) in RPMI 1640 medium at 37 °C for 40 min, followed by filtering through a nylon 200‐mesh filters to obtain the single cell suspensions. The lymphocytes from lymph nodes and spleens were obtained through mechanical disruption and then lysing the red blood cells with red blood cell lysis buffer (Biosharp, Hefei, China). In addition, 100 µL of EDTA‐anticoagulated blood was collected, washed with PBS, and lysed with red blood cell lysis buffer to obtain single blood cells. For flow cytometric analysis, the cells were then stained with anti‐CD45‐APC/Fire 750, anti‐CD45‐APC/Cy7, anti‐CD45‐FITC or anti‐CD45‐PercP/5.5, anti‐CD11c‐FITC or anti‐CD11c‐PE/Cy7, anti‐MHCII‐Percp/Cy5.5, anti‐H‐2K^b^‐SIINFEKL‐PE, anti‐CD103‐APC/Cy7, anti‐CD11b‐PE/Cy7 or anti‐CD11b‐Percp/Cy5.5, anti‐Ly6C‐FITC or anti‐Ly6C‐PE, anti‐Ly6G‐PerCP/Cyanine5.5, anti‐CCR2‐PE, anti‐F4/80‐PE/Cy7 or anti‐F4/80‐APC, anti‐CD80‐PE, anti‐CD86‐APC, anti‐CD3‐FITC or anti‐CD3‐PE, anti‐CD8‐PE/Cy7, anti‐CD4‐Percp/Cy5.5, anti‐CD69‐BV421, anti‐CD69‐Pacific Blue or anti‐CD69‐APC, and anti‐CD25‐APC antibodies for surface makers. For intracellular cytokine staining, the cells were treated with fixation buffer and intracellular staining permeabilization wash buffer, followed by re‐staining with anti‐TNF‐α‐BV421 or anti‐IFN‐γ‐PE antibody. For transcription factor staining, the cells were treated with a transcription factor buffer set (BD Pharmingen, cat. No562574) after surface staining and re‐stained with anti‐FoxP3‐PE antibody. For memory T cell analysis, the cells were stained with anti‐CD44‐PE and anti‐CD62L‐APC antibodies. For Ki67 staining, the cells were treated with 70% ethanol after surface staining and re‐stained with an anti‐Ki67‐APC antibody. All antibodies were diluted according to the instructions and incubated with the cells at room temperature for 30 min. The cells were analyzed by the CytoFLEX flow cytometry. The details on the multi‐color antibody combination for each cell type are shown in Table S1, Supporting Information. Gating strategies for flow cytometric analysis of different cell types involved in this study have been presented in Figures S24–S27, Supporting Information.

### Training of Monocytes/Macrophages in Blood Ex Vivo

In vitro training of primary monocytes/macrophages in blood was performed according to the method mentioned before.^[^ ^42^ ^]^ 100 µL of EDTA‐anticoagulated blood was washed with PBS and centrifuged at 300 g for 5 min. The cell pellets were lysed with 1000 µL red blood cell lysis buffer at room temperature for 2 min to obtain single cells. The blood cells were evenly seeded in 6‐well plates and then re‐stimulated with 100 ng mL^−1^ LPS or B16‐OVA cell supernatants at 37 °C for 24 h. The cells were harvested, washed with PBS, and then stained with anti‐CD45‐APC/Fire 750, anti‐CD11b‐FITC, anti‐Ly6C‐PE, anti‐F4/80‐APC, anti‐Ly6G‐Percp/Cy5.5, or anti‐CD11b‐Percp/Cy5.5, anti‐CD80‐PE antibodies for surface makers. For intracellular cytokine staining, the cells were treated with fixation buffer and intracellular staining permeabilization wash buffer, followed by re‐staining with anti‐TNF‐α‐BV421 antibody. The cells were analyzed by the CytoFLEX flow cytometry.

### Prophylactic and Therapeutic Antitumor Effects

For the prophylactic study, female C57BL/6 mice (6‐week‐old) were subcutaneously injected at the right flank with PBS, BG, OVA, BG + OVA, EcN, BG@EcN, OVA@EcN or BG/OVA@EcN at the OVA dosage of 40 µg, BG dosage of 40 µg, and EcN dosage of 3 × 10^8^ CFU in 100 µL PBS per mouse once every 3 days for four times. Third days after the final vaccination, the vaccinated mice were subcutaneously challenged with 6 × 10^5^ B16‐OVA cells at the left flank. The tumor volume was measured with a caliper every other day and calculated according to the following formula: *V* = (length × width^2^)/2. The mice were used for long‐term survival analysis. The mice were euthanized when the tumor volume reached 1500 mm^3^. For the therapeutic effect study, female C57BL/6 mice (6‐week‐old) were subcutaneously injected with 6 × 10^5^ B16‐OVA cells. When the tumor volume of B16‐OVA tumor‐bearing mice reached about 40 mm^3^, the mice were subcutaneously injected with PBS, BG, OVA, BG + OVA, EcN, BG@EcN, OVA@EcN, or BG/OVA@EcN at the OVA dosage of 40 µg, BG dosage of 40 µg, and EcN dosage of 3 × 10^8^ CFU in 100 µL PBS per mouse on days 6, 9, 12, and 17. The tumor volume was measured with a caliper every other day. On day 20, some of the mice were sacrificed, and the organs (heart, liver, spleen, lung, and kidney) were removed. The organs were fixed with 4% paraformaldehyde, sectioned, and stained with H&E. The other mice were used for long‐term survival analysis. Mice were euthanized when the tumor volumes reached 1500 mm^3^.

### Preparation and Characterization of Personalized BG/Ag@EcN

Tumors with a volume of about 300 mm^3^ were resected from 4T1 tumor‐bearing mice. The tumor tissues were then processed through mechanical disruption and digested by 0.8 mg mL^−1^ collagenase I at 37 °C for 30 min. The cells were filtered through nylon 200‐mesh filters, washed with PBS, and then treated with RBC lysis buffer to lyse RBCs. The harvested single 4T1 cells were suspended in cold H_2_O buffer containing protease inhibitor cocktail (Roche, cat. No. 04693159001), followed by freezing at liquid nitrogen, thawing at room temperature, and disrupting by using bath ultrasonic cleaner at a fixed frequency of 42 kHz and power of 100 W. The cell lysates were centrifuged at 18 000 g for 30 min to collect cell membranes. The protein concentration of the membranes was quantified using a BCA protein assay kit. The personalized BG/Ag@EcN was obtained according to the same protocol except that using tumor cell membranes as tumor antigen. The zeta potentials of BG/Ag@EcN were measured with a Zetasizer Nano ZS90 (Malvern, UK).

### Anti‐Recurrence Effects of BG/Ag@EcN after Surgery

Orthotopic 4T1 tumor‐bearing mice were constructed by inoculating 4T1 cells (3 × 10^5^ cells per mouse) into the right fourth breast fat pad of female BALB/c mice. When tumor size reached about 300 mm^3^, 90% of the tumors were resected. The mice were subcutaneously injected with PBS, BG, free Ag, BG + Ag, EcN, BG@EcN, Ag@EcN, or BG/Ag@EcN at the Ag dosage of 40 µg, BG dosage of 40 µg, and EcN 3 × 10^8^ CFU in 100 µL PBS per mouse on days 3, 6, 9, and 14 after surgery. Tumor volumes and body weights of the mice were recorded every 2 days. The mice were used for long‐term survival analysis. Mice were euthanized when the tumor volumes reached 1500 mm^3^.

### Statistical Analysis

All data were presented as mean ± SD with at least three independent experiments. One‐way analysis of variance (ANOVA) followed by Tukey's honest significant difference (HSD) post‐hoc test, or two‐way ANOVA with Bonferroni correction was used to perform the statistical comparison between multiple groups. An unpaired two‐tailed Student's *t*‐test was used to determine the significance between the two groups. Detailed sample sizes are shown in figure legends. GraphPad Prism 7.0 software (GraphPad Software, La Jolla, USA) was utilized for data analysis. In terms of all statistical tests, *p‐*value < 0.05 was considered statistically significant. n.s: no significance, *p < 0.05, **p < 0.01, and ***p < 0.001.

## Conflict of Interest

The authors declare no conflict of interest.

## Author Contributions

Z.C. and T.Y. contributed equally to this work. L.G., T.Y., Z.C., and X.Y. designed the project. Z.C., T.Y., Z.W., X.Z., X.L., J.Q., J.L., and J.H. performed the experiments. Z.C., T.Y., X.Y., and L.G. analyzed and interpreted the data, and wrote the manuscript.

## Supporting information

Supporting information

## Data Availability

The data that support the findings of this study are available from the corresponding author upon reasonable request.
