# Supplementary material for: Engineered Probiotic‐Based Personalized Cancer Vaccine Potentiates Antitumor Immunity through Initiating Trained Immunity
Source: Adv Sci (Weinh). 2023 Nov 27;11(3):2305081. doi: 10.1002/advs.202305081 (PMC10797439; doi:10.1002/advs.202305081)
Supplement: Supplementary file 1 — Supporting information [file ADVS-11-2305081-s001.pdf]

ADVANCED SCIENCE
Open Access

Supporting Information

for *Adv. Sci.*, DOI 10.1002/advs.202305081

Engineered Probiotic-Based Personalized Cancer Vaccine Potentiates Antitumor Immunity through Initiating Trained Immunity

Zhaoxia Chen, Tuying Yong, Zhaohan Wei, Xiaoqiong Zhang, Xin Li, Jiaqi Qin, Jianye Li, Jun Hu, Xiangliang Yang* and Lu Gan**

Supporting Information

Engineered probiotic-based personalized cancer vaccine potentiates antitumor immunity through initiating trained immunity

Zhaoxia Chen[1,#], Tuying Yong[1,2,3,#,*], Zhaohan Wei[1], Xiaoqiong Zhang[1], Xin Li[1], Jiaqi Qin[1], Jianye Li[1], Jun Hu[1,2,3], Xiangliang Yang[1,2,3,*], Lu Gan[1,2,3,*]

[1]National Engineering Research Center for Nanomedicine, College of Life Science and Technology, Huazhong University of Science and Technology, Wuhan 430074, China

[2]Key Laboratory of Molecular Biophysics of the Ministry of Education, College of Life Science and Technology, Huazhong University of Science and Technology, Wuhan 430074, China

[3]Hubei Key Laboratory of Bioinorganic Chemistry and Materia Medica, Huazhong University of Science and Technology, Wuhan 430074, China

[#]Zhaoxia Chen and Tuying Yong contributed equally to this work.

[*]Correspondence should be addressed to: lugan@mail.hust.edu.cn, yangxl@mail.hust.edu.cn, yongty2018@hust.edu.cn

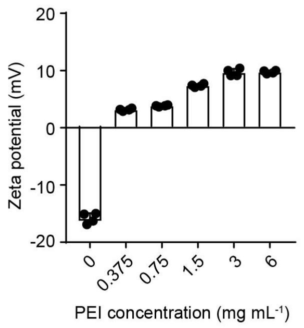

Figure S1. Zeta potential of EcN after PEI coating. EcN was modified with different concentrations of PEI, and their zeta potentials were determined by DLS analysis. Data are presented as means ± s.d. (n = 4).

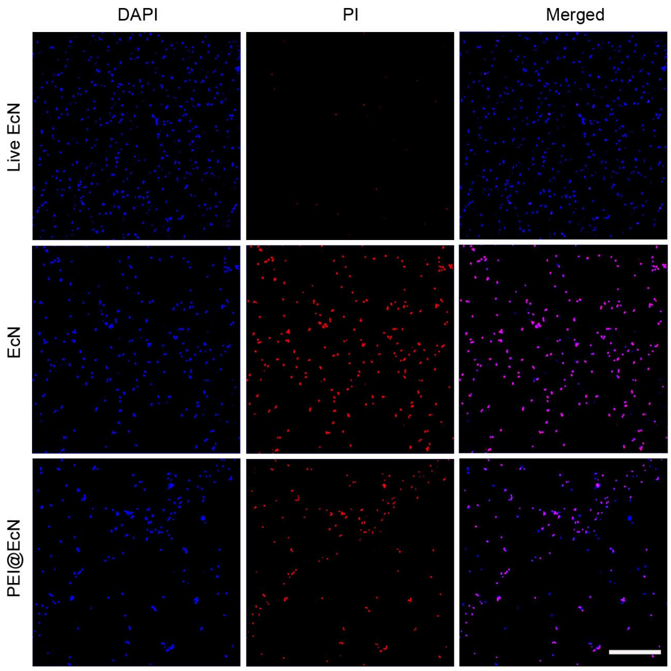

Figure S2. Confirmation of the inactivation of EcN by high temperature. Live EcN, EcN inactivated high temperature and PEI@EcN were stained with DAPI/PI and then observed by confocal microscopy. Scale bar: 30 μm.

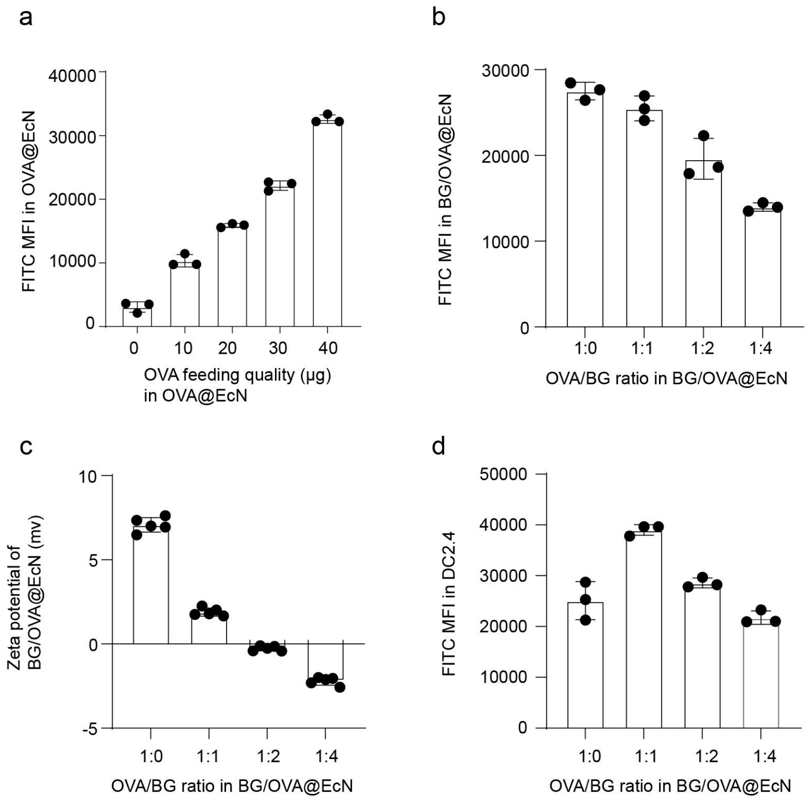

Figure S3. Optimization of the preparation of BG/OVA@EcN. a) FITC MFI of OVA@EcN after incubation of 3×10^{8} CFU of EcN with different contents of OVA-FITC in 200 μL^{-1} PBS by flow cytometry. Data are presented as means ± s.d. (n = 3). b-c) FITC MFI (b) and zeta potential (c) of BG/OVA@EcN after incubation of 3×10^{8} CFU of EcN, 40 μg OVA and BG with the different OVA/BG mass ratios in 200 μL PBS by flow cytometry and DLS analysis, respectively. Data are presented as means ± s.d. (n = 3). d) FITC MFI in DC2.4 cells after DC2.4 cells were treated with BG/OVA@EcN at the concentration of 3×10^{7} CFU mL^{-1} EcN with the different OVA/BG mass ratios for 24 h by flow cytometry. Data are presented as means ± s.d. (n = 3).

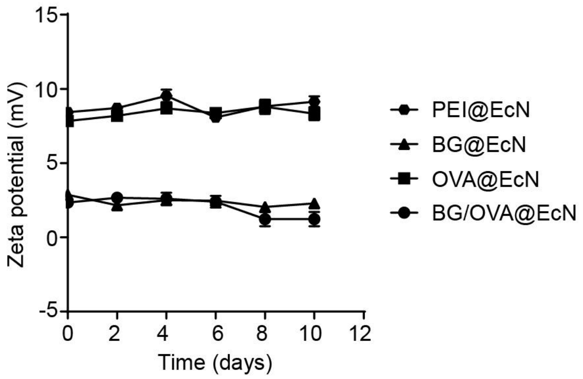

Figure S4. Stability of BG/OVA@EcN. PEI@EcN, BG@EcN, OVA@EcN and BG/OVA@EcN were incubated in PBS for different time intervals, and their Zeta potentials were determined by DLS analysis. Data are presented as means ± s.d. (n = 3).

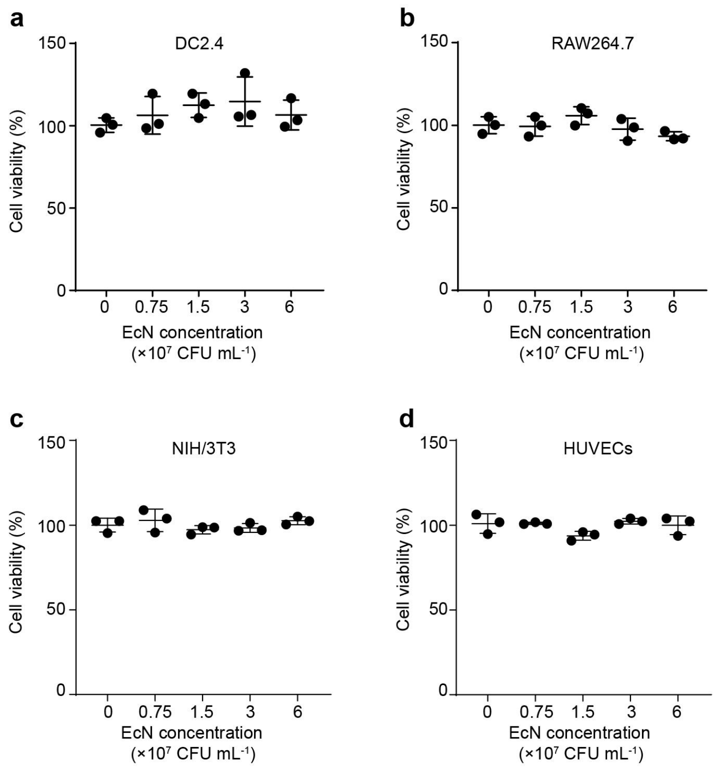

Figure S5. Biocompatibility of BG/OVA@EcN. a-d) Cell viability of DC2.4 (a), RAW264.7 (b), NIH/3T3 (c) and HUVECs (d) cells after treatment with BG/OVA@EcN at the different concentrations of EcN for 12 h by CCK-8 assay. Data are presented as means ± s.d. (n = 3).

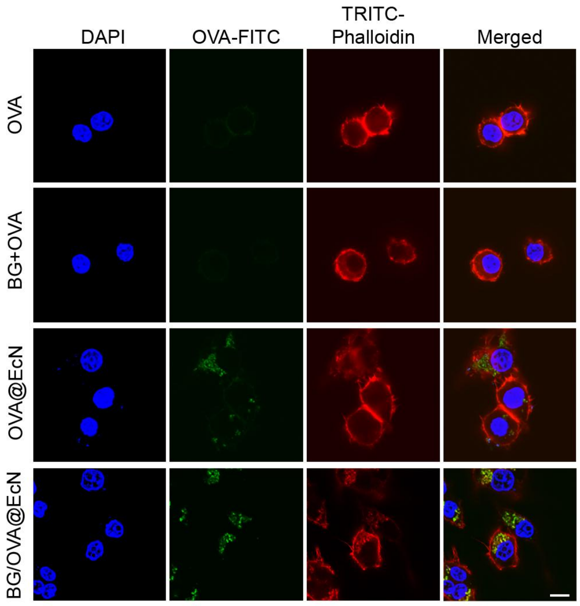

Figure S6. Efficient phagocytosis of BG/OVA@EcN by RAW264.7 macrophages. RAW264.7 cells were treated with free OVA, BG + OVA, OVA@EcN or BG/OVA@EcN (OVA was conjugated with FITC) at the concentration of 3×10^7 CFU mL^{-1} EcN, 4 μg mL^{-1} OVA and 4 μg mL^{-1} BG for 4 h, and then observed by confocal microscopy. RAW264.7 cells were labeled with TRITC-conjugated phalloidin. Scale bar: 10 μm.

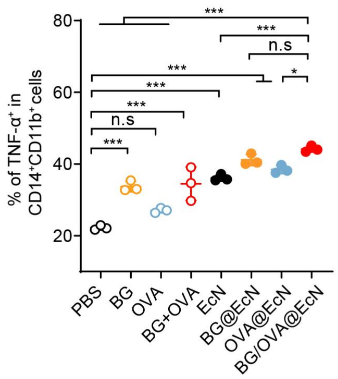

Figure S7. BG/OVA@EcN-mediated training of human-derived monocytes/macrophages *in vitro*. THP-1 cells were treated with PBS, BG, OVA, BG + OVA, EcN, BG@EcN, OVA@EcN, BG/OVA@EcN at the concentration of 3×10^7 CFU mL^{-1} EcN, 4 μg mL^{-1} OVA and 4 μg mL^{-1} BG for 12 h, followed by resting for 5 days and re-stimulating with 100 ng mL^{-1} LPS for 24 h by flow cytometry. Data are presented as means ± s.d. (n = 3). *P*-values are calculated using one-way ANOVA followed by Tukey's HSD post hoc test. * $P < 0.05$, ***$P < 0.001$.

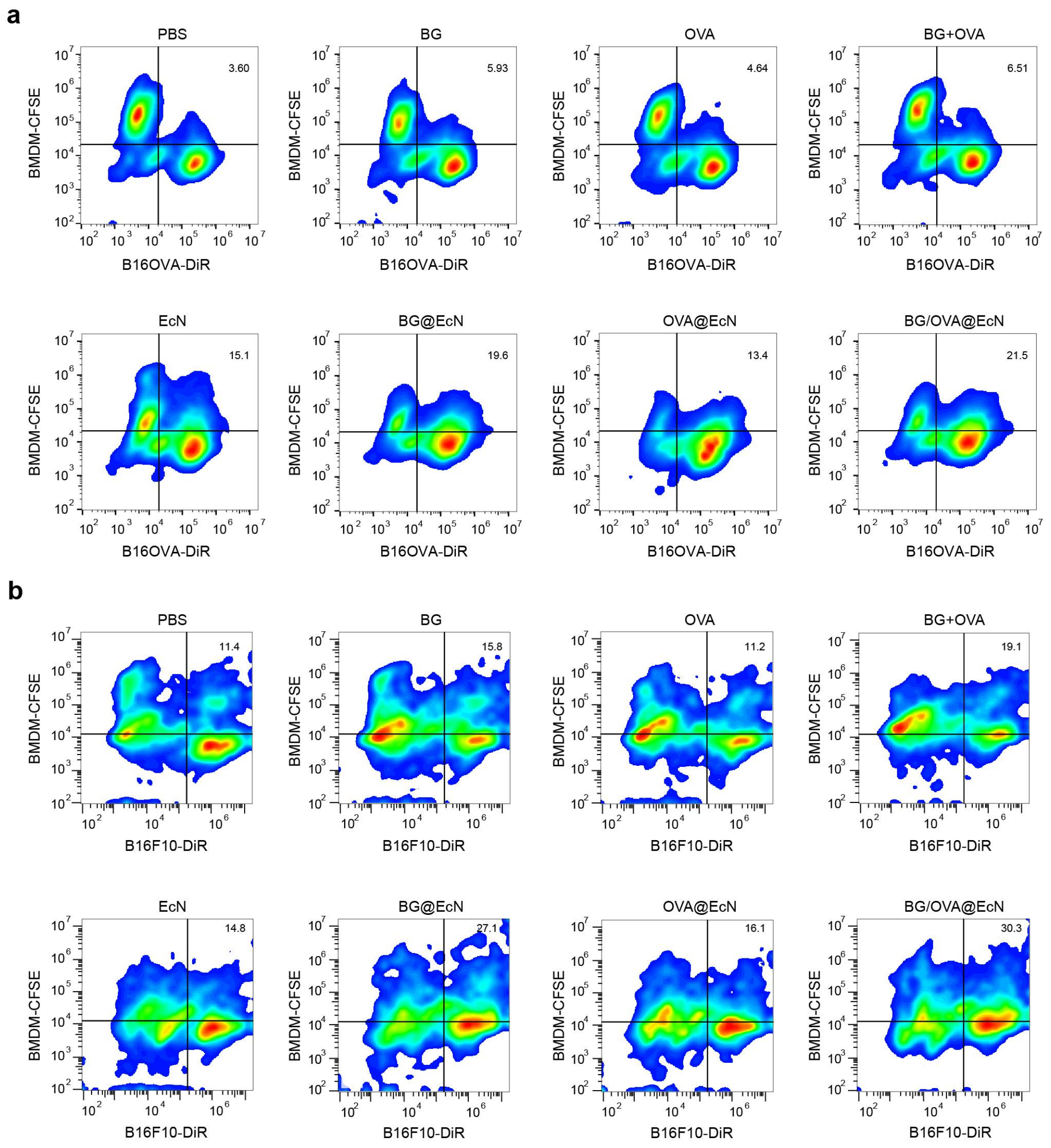

Figure S8. Phagocytosis of B16-OVA and B16F10 cells by BG/OVA@EcN-trained macrophages. a, b) Representative flow cytometric analysis of DiR-labeled B16-OVA (a) and B16F10 cells (b) by CSFE-labeled BMDMs after BMDMs were treated with PBS, BG, OVA, BG + OVA, EcN, BG@EcN, OVA@EcN, BG/OVA@EcN at the concentration of 3×10^{7} CFU mL^{-1} EcN, 4 μg mL^{-1} OVA and 4 μg mL^{-1} BG for 12 h, followed by resting for 5 days and then co-culturing with DiR-labeled B16-OVA cells at the ratio of 1:1 for 4 h.

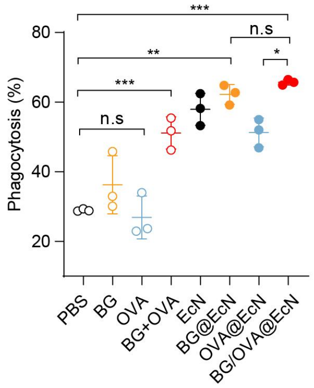

Figure S9. Phagocytosis ratios of DiR-labeled B16F10 cells by CSFE-labeled BMDMs after BMDMs were treated with PBS, BG, OVA, BG + OVA, EcN, BG@EcN, OVA@EcN, BG/OVA@EcN at the concentration of 3×10^7 CFU mL^{-1} EcN, 4 μg mL^{-1} OVA and 4 μg mL^{-1} BG for 12 h, followed by resting for 5 days and then co-culturing with DiR-labeled B16F10 cells at the ratio of 1:1 for 4 h by flow cytometry. Phagocytosis was calculated as the percentage of DiR^+CSFE^+ cells among $CFSE^+$ BMDMs. Data are presented as means ± s.d. (n = 3). *P*-values are calculated using one-way ANOVA followed by Tukey's HSD post hoc test.* $P < 0.05$, ** $P < 0.01$, *** $P < 0.001$.

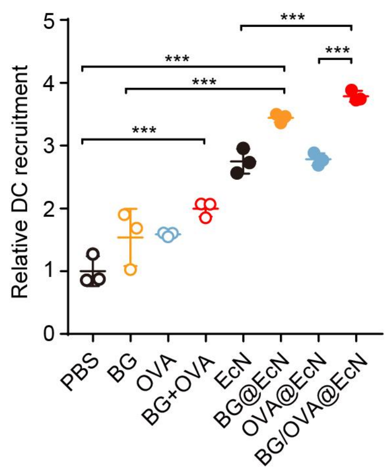

Figure S10. Relative DC recruitment by the supernatants of BG/OVA@EcN-trained macrophages. BMDCs isolated from C57BL/6 mice were seeded in the top chambers and the supernatants of BMDMs pretreated with PBS, BG, OVA, BG + OVA, EcN, BG@EcN, OVA@EcN or BG/OVA@EcN at the concentration of 3×10^7 CFU mL^{-1} EcN, 4 μg mL^{-1} OVA and 4 μg mL^{-1} BG for 12 h were added in the bottom chambers. After 8 h, the numbers of $CD11c^+$ cells in the bottom chambers were determined by flow cytometry. Data are presented as means ± s.d. (n = 3). *P*-values are calculated using one-way ANOVA followed by Tukey's HSD post hoc test. ***$P < 0.001$.

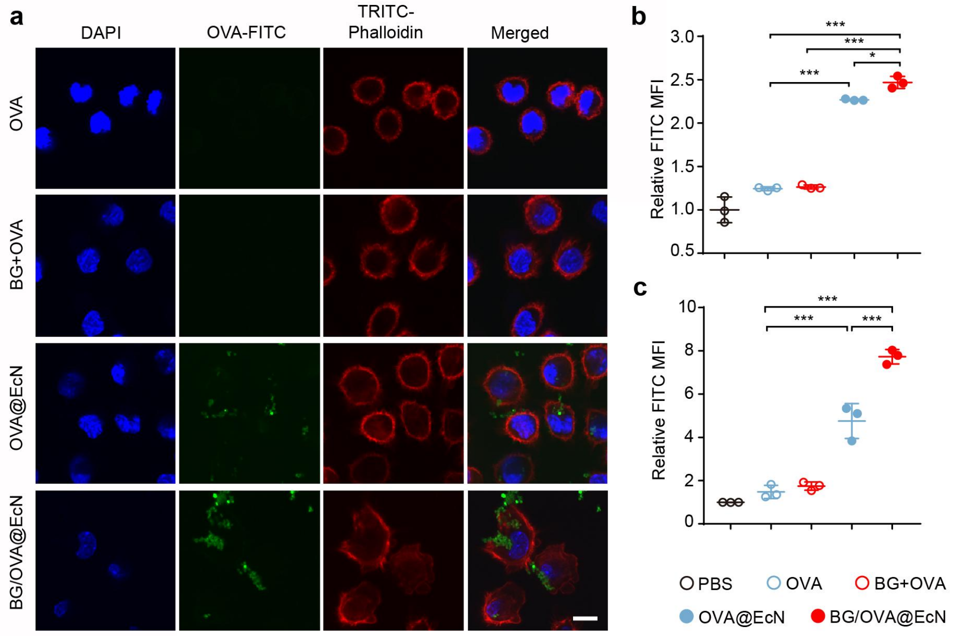

Figure S11. Efficient phagocytosis of BG/OVA@EcN by DCs. a) Confocal microscopic images of DC2.4 cells after treatment with free OVA, BG + OVA, OVA@EcN or BG/OVA@EcN (OVA was conjugated with FITC) at the concentration of 3×10^7 CFU mL^{-1} EcN, 4 μg mL^{-1} OVA and 4 μg mL^{-1} BG for 4 h. DC2.4 cells were labeled with TRITC-conjugated phalloidin. Scale bar: 10 μm. b-c) Relative FITC MFI in DC2.4 cells (b) and BMDCs (c) after treatment indicated in (a) by flow cytometry. Data are presented as means ± s.d. (n = 3). *P*-values are calculated using one-way ANOVA followed by Tukey's HSD post hoc test. * $P < 0.05$, *** $P < 0.001$.

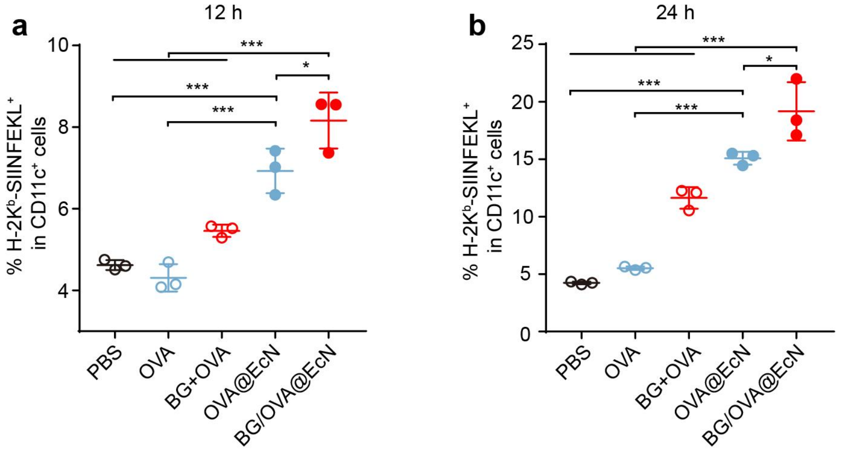

Figure S12. Efficient antigen processing and presentation of BG/OVA@EcN by BMDCs. a-b) Percentages of H-2K^b-SIINFEKL$^+$ cells in CD11c$^+$ cells after BMDCs were treated with PBS, OVA, BG + OVA, OVA@EcN or BG/OVA@EcN at the concentration of 3 × 10^7 CFU mL^{-1} EcN, 4 μg mL^{-1} OVA and 4 μg mL^{-1} BG for 12 h (a) or 24 h (b) by flow cytometry. Data are presented as means ± s.d. (n = 3). *P*-values are calculated using one-way ANOVA followed by Tukey's HSD post hoc test. * $P <$ 0.05, *** $P < 0.001$.

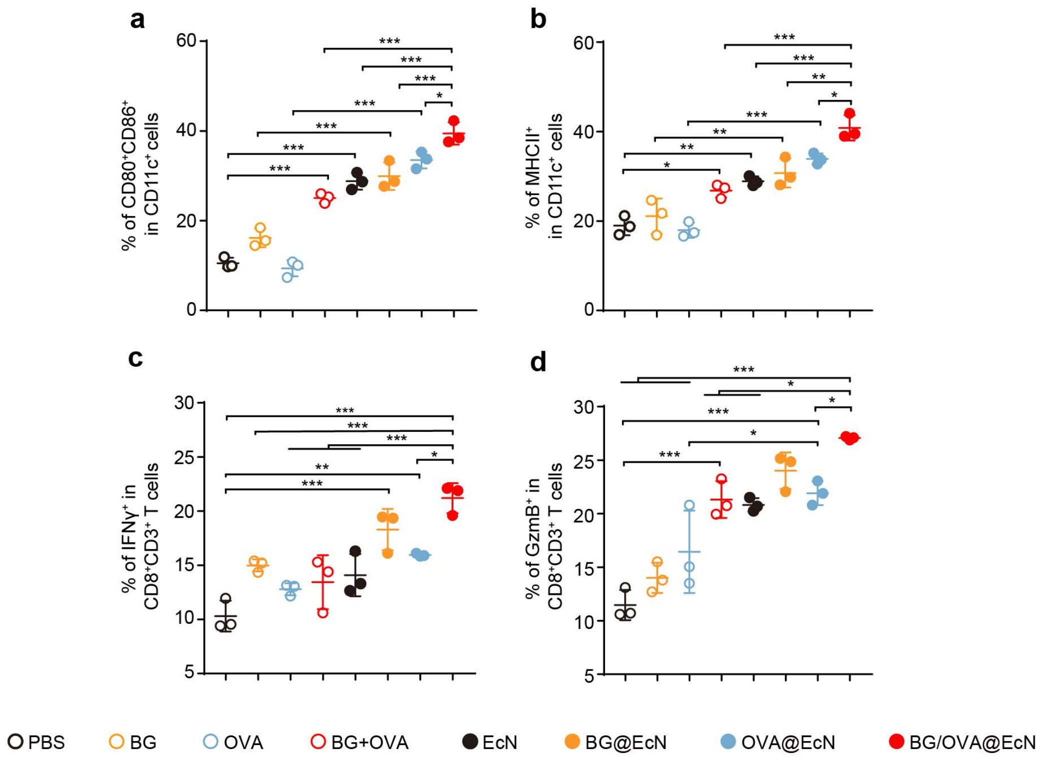

Figure S13. BG/OVA@EcN-triggered effcient DC maturation and CD8$^+$ T cell activation. a-b) Percentages of CD80$^+$CD86$^+$ cells (a) and MHCII$^+$ cells (b) in CD11c$^+$ cells after BMDCs were treated with PBS, BG, OVA, BG + OVA, EcN, BG@EcN, OVA@EcN, BG/OVA@EcN at the concentration of 3×10^7 CFU mL^{-1} EcN, 4 µg mL^{-1} OVA and 4 µg mL^{-1} BG for 12 h by flow cytometry. Data are presented as means ± s.d. (n = 3). c-d) Percentages of IFNγ$^+$ (c) and GzmB$^+$ cells (d) in CD3$^+$CD8$^+$ T cells after CD8$^+$ T cells were incubated with the above-matured BMDCs for 3 days by flow cytometry. Data are presented as means ± s.d. (n = 3). *P*-values are calculated using one-way ANOVA followed by Tukey's HSD post hoc test. * $P < 0.05$, ** $P < 0.01$, *** $P < 0.001$.

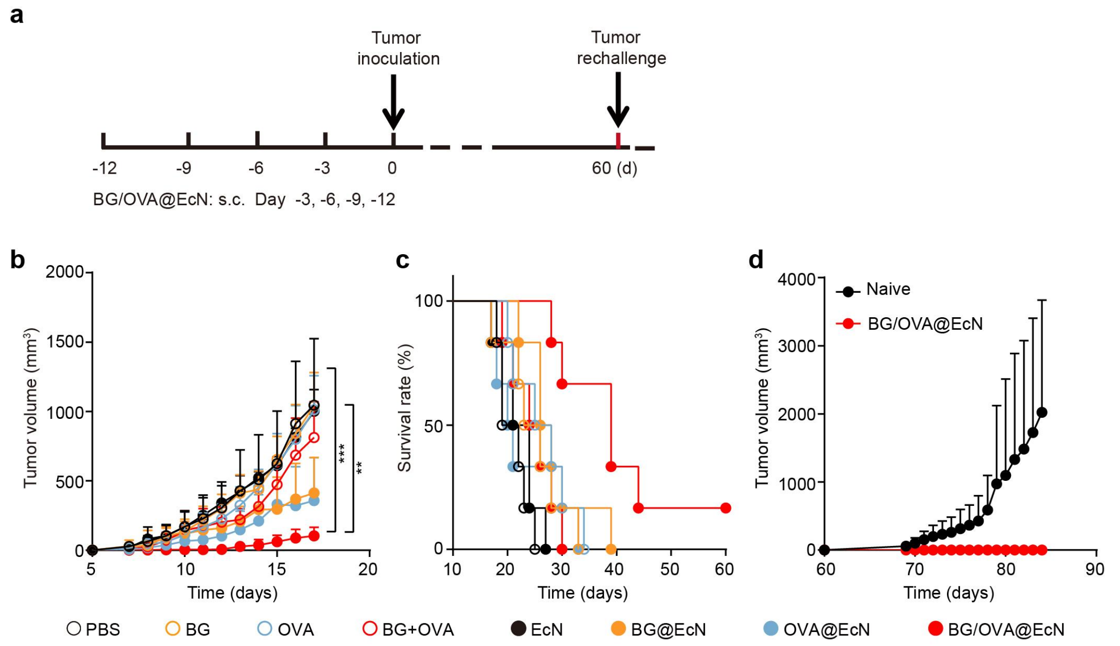

Figure S14. Prophylactic effects of BG/OVA@EcN against B16-OVA melanoma. a) Schematic schedule for the prophylactic experiments in subcutaneous B16-OVA tumor-bearing mice. b) Tumour growth curves of B16-OVA tumor-bearing mice after subcutaneous injection of PBS, BG, OVA, BG + OVA, EcN, BG@EcN, OVA@EcN or BG/OVA@EcN at the OVA dosage of 40 μg, BG dosage of 40 μg and EcN dosage of 3×10^8 CFU per mouse as indicated in (a). Data are presented as means ± s.d. (n = 6). c) Kaplan-Meier survival plots of B16-OVA tumor-bearing mice after treatment indicated in a. (n = 6). d) Tumor growth curve of B16-OVA tumor-bearing mice after re-challenge with B16-OVA cells in naïve mice or BG/OVA@EcN-treated tumor-free mice. Data are presented as means ± s.d. (n = 1 for BG/OVA@EcN-treated mice, n = 5 for naïve mice). *P*-values are calculated using one-way ANOVA followed by Tukey's HSD post hoc test. ** $P < 0.01$, *** $P < 0.001$.

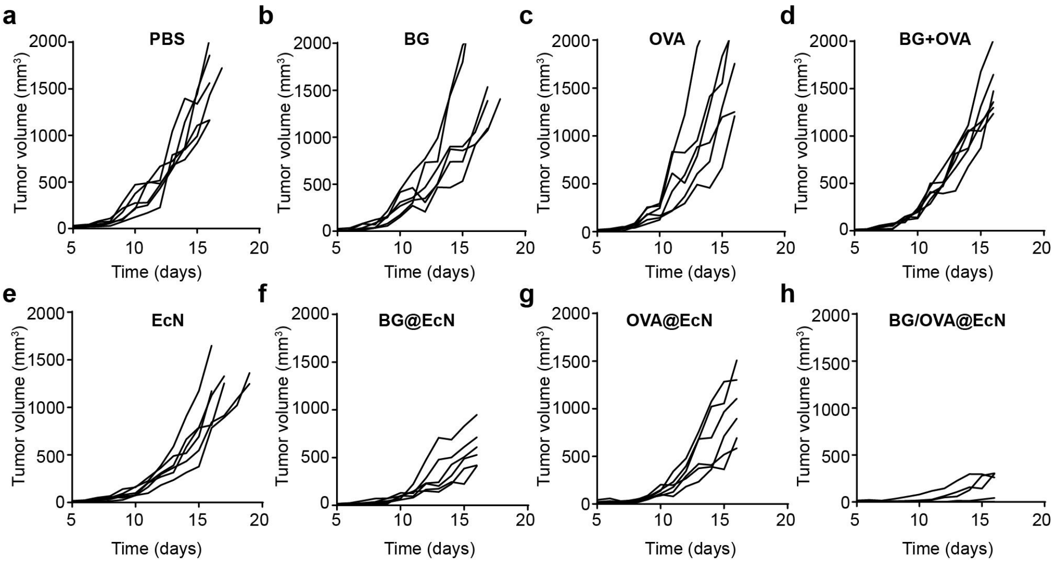

Figure S15. Anticancer activity of BG/OVA@EcN in subcutaneous B16-OVA tumor-bearing mice. a-h) Individual tumour growth curves of B16-OVA tumor-bearing mice after subcutaneous injection of PBS (a), BG (b), OVA (c), BG + OVA (d), EcN (e), BG@EcN (f), OVA@EcN (g) or BG/OVA@EcN (h) at the OVA dosage of 40 μg, BG dosage of 40 μg and EcN dosage of 3×10^8 CFU per mouse as indicated in Figure 6a.

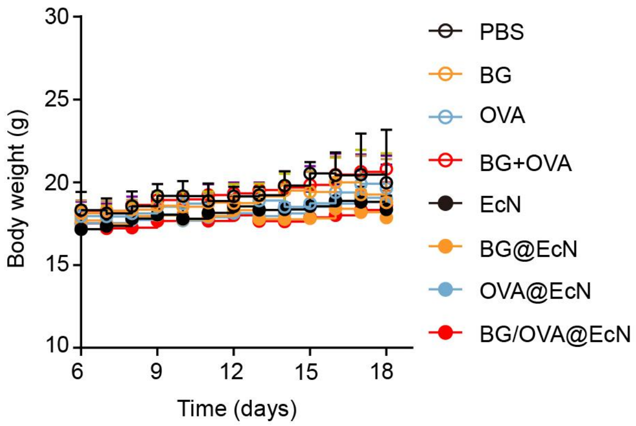

Figure S16. Body weight of B16-OVA tumor-bearing mice after treatment with BG/OVA@EcN. B16-OVA tumor-bearing mice were subcutaneously injected with PBS, BG, OVA, BG + OVA, EcN, BG@EcN, OVA@EcN or BG/OVA@EcN at the OVA dosage of 40 μg, BG dosage of 40 μg and EcN dosage of 3×10^8 CFU per mouse as indicated in Figure 6a, and the body weight was measured every other day. Data are presented as means ± s.d. (n = 6).

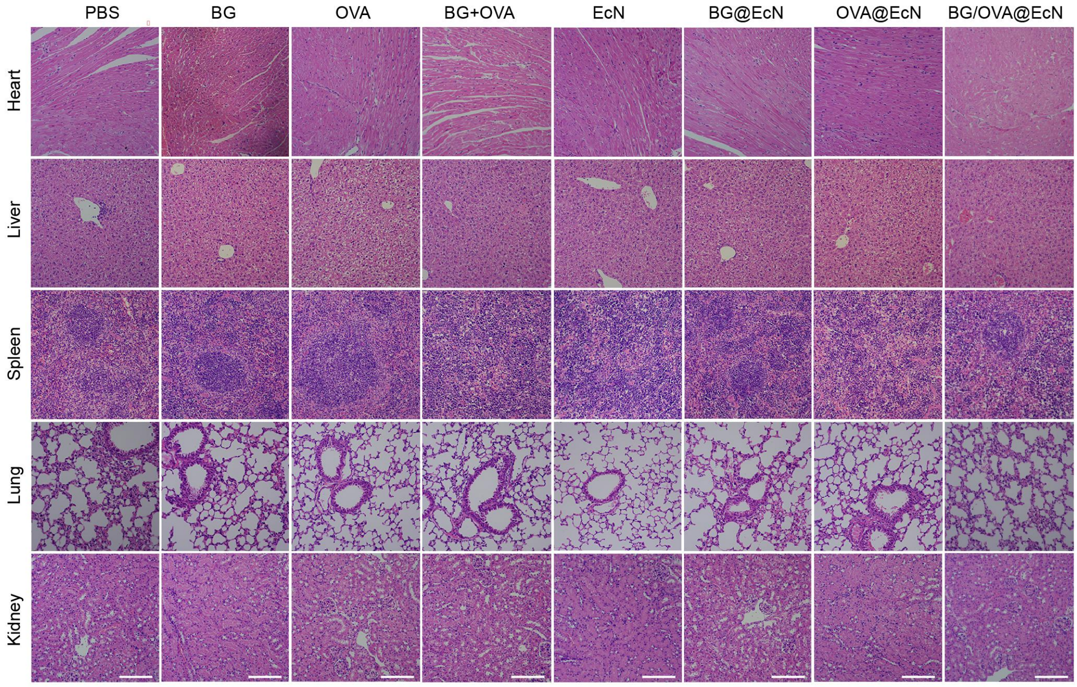

Figure S17. H&E staining of major organs (including heart, liver, spleen, lung and kidney) of B16-OVA tumor-bearing mice at 20 days after subcutaneous injection of PBS, BG, OVA, BG + OVA, EcN, BG@EcN, OVA@EcN or BG/OVA@EcN at the OVA dosage of 40 μg, BG dosage of 40 μg and EcN dosage of 3×10^8 CFU per mouse as indicated in Figure 6a. Scale bars: 50 μm.

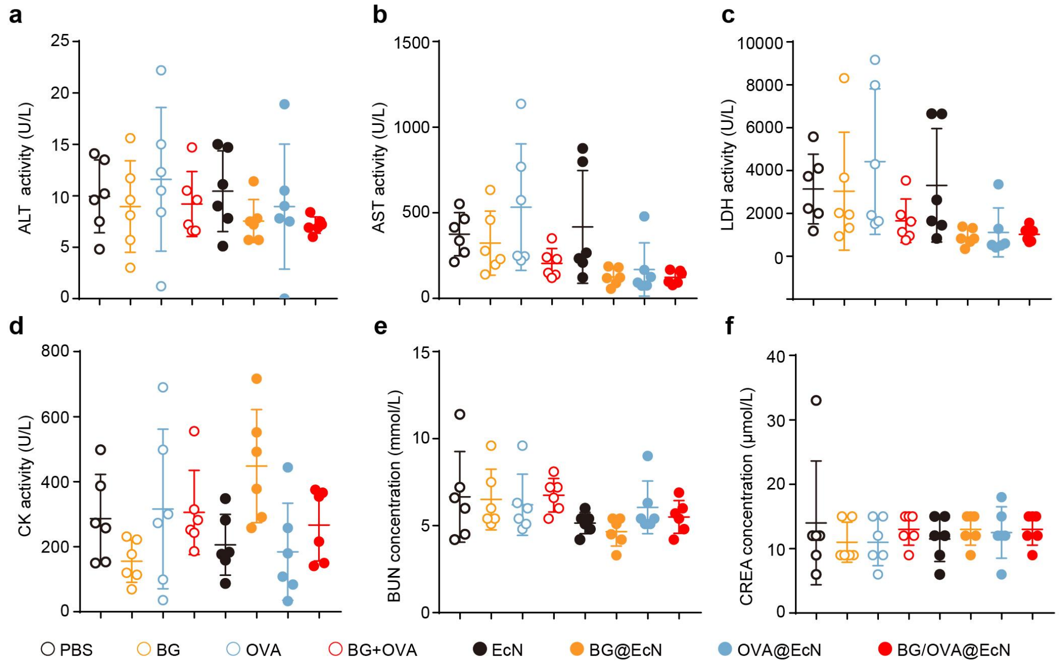

Figure S18. Serological analysis of B16-OVA tumor-bearing mice after treatment with BG/OVA@EcN. a-f) The serological analysis of alanine aminotransferase (ALT, a), aspartate aminotransferase (AST, b), lactate dehydrogenase (LDH, c), creatine kinase (CK, d), blood urea nitrogen (BUN, e) and creatinine (CREA, f) in B16-OVA tumor-bearing mice at 20 days after subcutaneous injection of PBS, BG, OVA, BG + OVA, EcN, BG@EcN, OVA@EcN or BG/OVA@EcN at the OVA dosage of 40 μg, BG dosage of 40 μg and EcN dosage of 3×10^8 CFU per mouse as indicated in Figure 6a. Data are presented as means ± s.d. (n = 6).

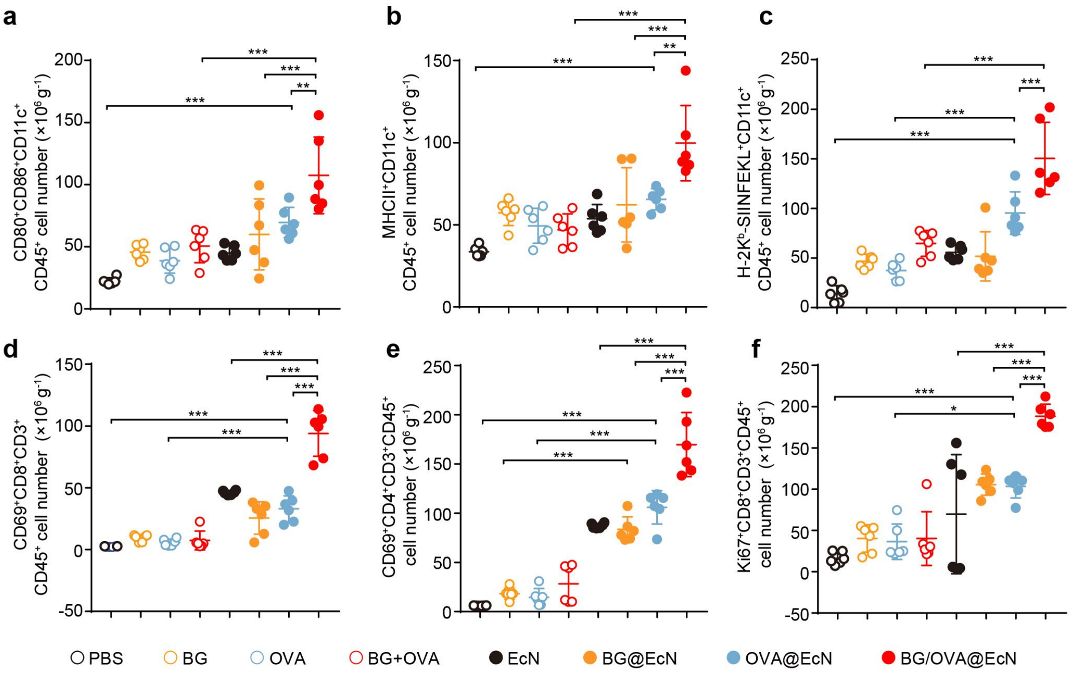

Figure S19. BG/OVA@EcN-improved immune microenvironment in draining lymph nodes of B16-OVA tumor-bearing mice. a-f) The numbers of $CD80^{+}CD86^{+}$ DCs (a), $MHCII^{+}$ DCs (b), H-$2K^{b}$-$SIINFEKL^{+}$ DCs (c), $CD69^{+}CD8^{+}$ T cells (d), $CD69^{+}CD4^{+}$ T cells (e) and $Ki67^{+}CD8^{+}$ T cells (f) in draining lymph nodes of B16-OVA tumor-bearing mice at 20 days after subcutaneous injection of PBS, BG, OVA, BG + OVA, EcN, BG@EcN, OVA@EcN or BG/OVA@EcN at the OVA dosage of 40 μg, BG dosage of 40 μg and EcN dosage of 3×10^{8} CFU per mouse as indicated in Figure 6a. Data are presented as means ± s.d. (n = 6). *P*-values are calculated using one-way ANOVA followed by Tukey's HSD post hoc test. **P*<0.05, ***P*<0.01, ****P*<0.001.

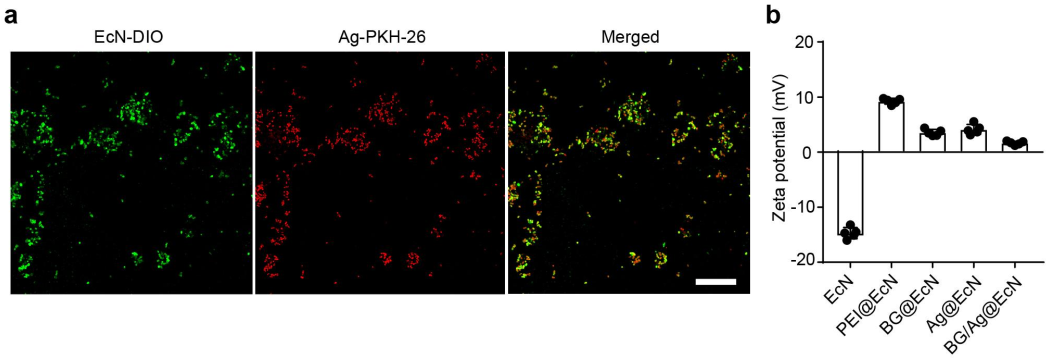

Figure S20. Successful construction of BG/Ag@EcN. a) Representative confocal microscopic images of BG/Ag@EcN in which Ag was labeled with PKH-26 and EcN was labeled with DIO. Scale bar: 20 μm. b) Zeta potential of inactivated EcN, PEI@EcN, BG@EcN, Ag@EcN and BG/Ag@EcN by DLS analysis, respectively. Data are presented as means ± s.d. (n = 5).

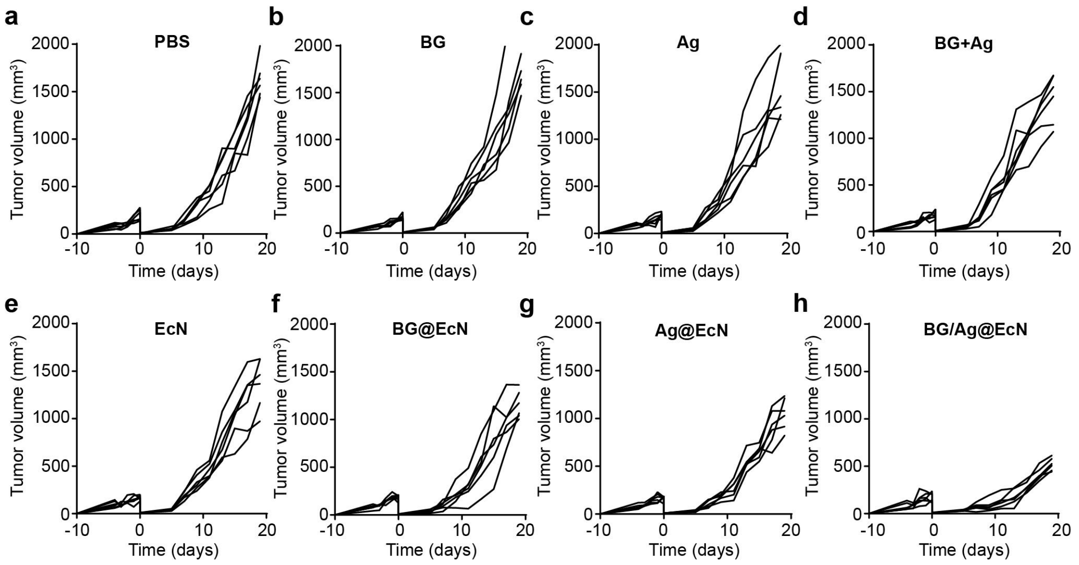

Figure S21. Anti-recurrence activity of BG/Ag@EcN in 4T1 tumor-bearing mice undergoing surgical tumor resection. a-h) Individual tumor growth curves of 4T1 tumor-bearing mice undergoing surgical tumor resection after subcutaneous injection of PBS (a), BG (b), Ag (c), BG + Ag (d), EcN (e), BG@EcN (f), Ag@EcN (g) or BG/Ag@EcN (h) at the Ag dosage of 40 μg, BG dosage of 40 μg and EcN 3 × 10^8 CFU per mouse as indicated in Figure 7a.

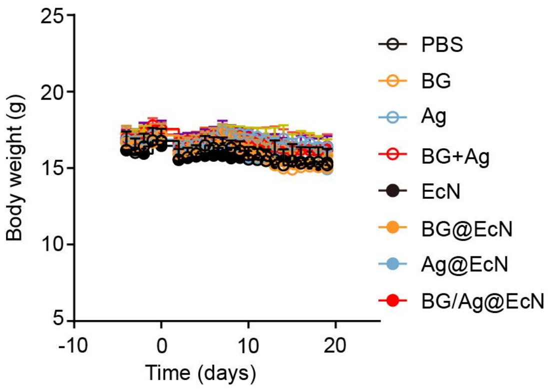

Figure S22. Body weight of 4T1 tumor-bearing mice undergoing tumor resection after treatment with BG/Ag@EcN. Orthotopic 4T1 tumor-bearing mice undergoing tumor resection were subcutaneously injected with PBS, BG, Ag, BG + Ag, EcN, BG@EcN, Ag@EcN or BG/Ag@EcN at the Ag dosage of 40 µg, BG dosage of 40 µg and EcN 3 × 10^8 CFU per mouse as indicated in Figure 7a， and the body weight was measured every other day. Data are presented as means ± s.d. (n = 6).

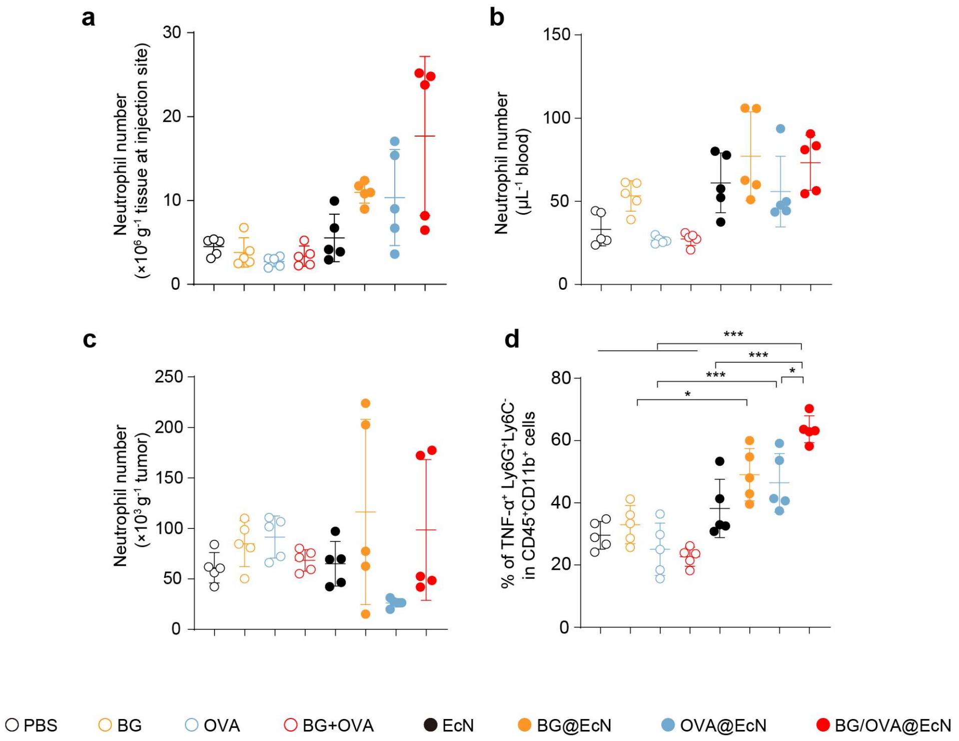

Figure S23. Effects of BG/OVA@EcN on the neutrophils. a-b) Numbers of neutrophils at the injection sites (a) and blood (b) of healthy C57BL/6 mice at 3 days after subcutaneous injection of PBS, BG, OVA, BG + OVA, EcN, BG@EcN, OVA@EcN or BG/OVA@EcN at the OVA dosage of 40 μg, BG dosage of 40 μg and EcN dosage of 3×10^8 CFU per mouse. Data are presented as means ± s.d. (n = 5). c) Numbers of neutrophils in tumor tissues of B16-OVA tumor-bearing mice after treatment indicated in Figure 6a. Data are presented as means ± s.d. (n = 5). d) Percentages of TNF-α^+ neutrophils after the blood cells from C57BL/6 mice treated as a-b was re-stimulated with 100 ng mL^{-1} LPS for 24 h. Data are presented as means ± s.d. (n = 5). *P*-values are calculated using one-way ANOVA followed by Tukey's HSD post hoc test. ** $P < 0.01$, *** $P < 0.001$.

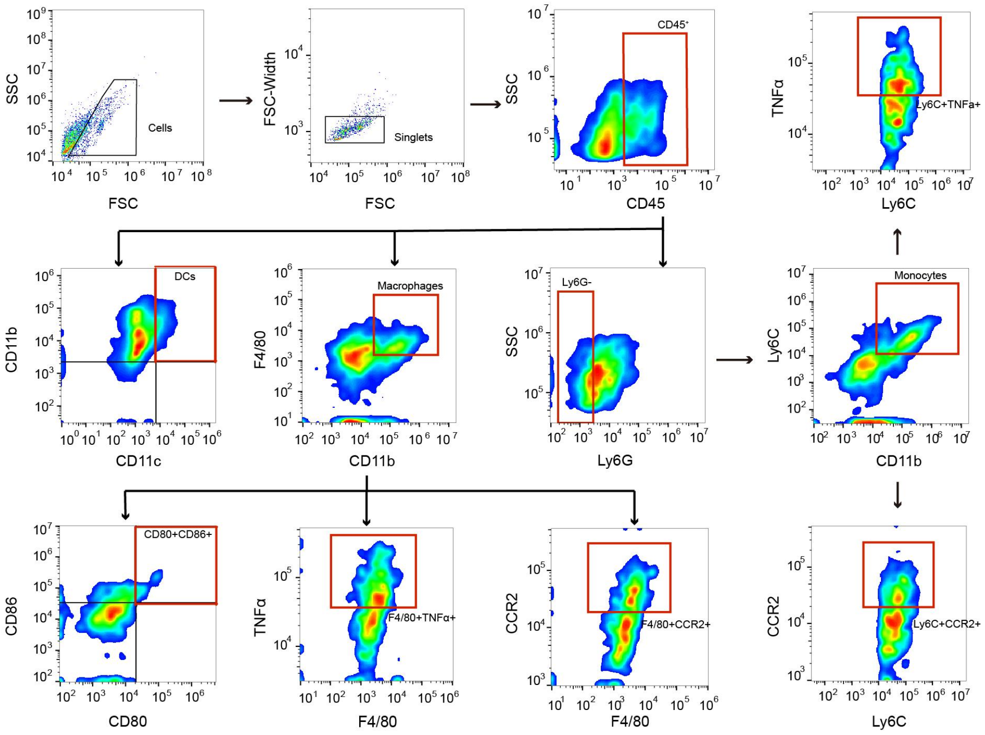

Figure S24. Gating strategies for identifying macrophages (CD45$^+$CD11b$^+$F4/80$^+$), matured macrophages (CD45$^+$CD11b$^+$F4/80$^+$CD80$^+$CD86$^+$), TNF-α$^+$ macrophages (CD45$^+$CD11b$^+$F4/80$^+$TNF-α$^+$), monocytes (CD45$^+$CD11b$^+$Ly6G$^-$Ly6C$^+$), TNF-α$^+$ monocytes (CD45$^+$CD11b$^+$F4/80$^+$TNF-α$^+$), CCR2$^+$ monocytes (CD45$^+$CD11b$^+$Ly6G$^-$Ly6C$^+$CCR2$^+$), CCR2$^+$ macrophages (CD45$^+$CD11b$^+$F4/80$^+$CCR2$^+$), total DCs (CD45$^+$CD11b$^+$CD11c$^+$ cells) at the subcutaneous injection sites of healthy mice presented in Figure 3d-i.

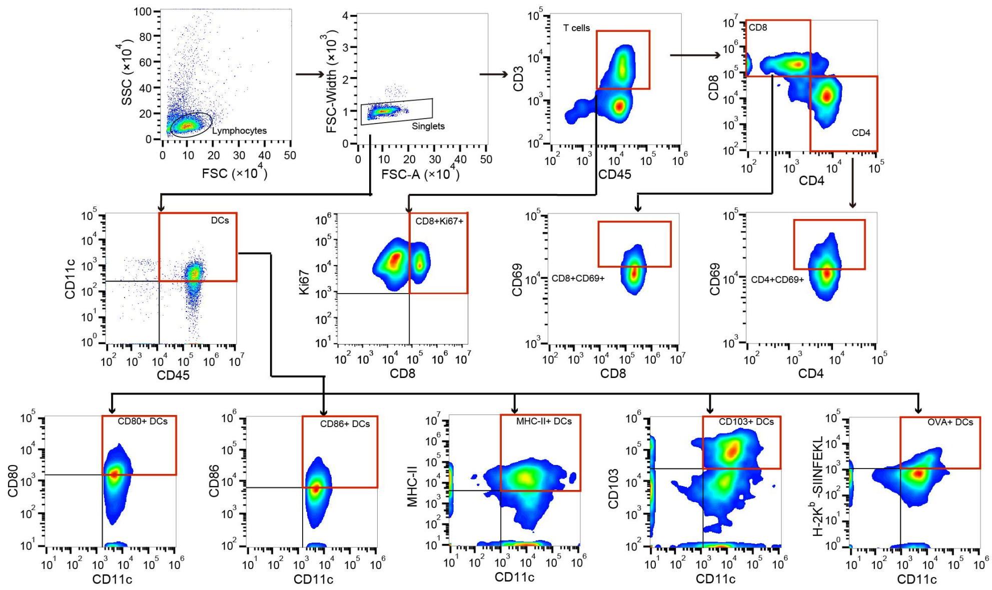

Figure S25. Gating strategies for identifying matured DCs (CD45^{+}CD11C^{+}CD80^{+}, CD45^{+}CD11C^{+}CD86^{+} and CD45^{+}CD11C^{+}MHCII^{+} cells), migratory DCs (CD45^{+}CD11C^{+}CD103^{+} cells), H-2k^{b}-SIINFEKL^{+}DCs (CD45^{+}CD11C^{+}H-2k^{b}-SIINFEKL^{+} cells), CD3^{+} T cells (CD45^{+}CD3^{+} cells), CD8^{+} T cells (CD45^{+}CD3^{+}CD8^{+} cells), CD4^{+} T cells (CD45^{+}CD3^{+}CD4^{+} cells), CD8^{+}CD69^{+} T cells (CD45^{+}CD3^{+}CD8^{+}CD69^{+} cells), CD4^{+}CD69^{+} T cells (CD45^{+}CD3^{+}CD4^{+}CD69^{+} cells) in lymph nodes or subcutaneous injection sites of mice presented in Figure 4c-n and Figure S19a-f.

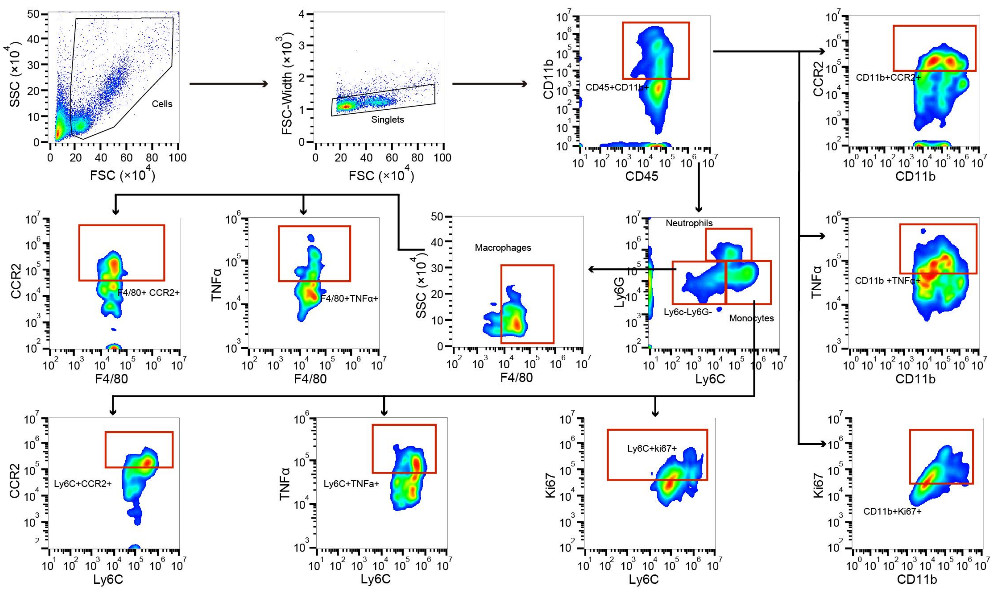

Figure S26. Gating strategies for identifying myeloid cells (CD45$^+$CD11b$^+$ cells), monocytes (CD45$^+$CD11b$^+$Ly6G$^-$Ly6C$^+$ cells), macrophages (CD45$^+$CD11b$^+$Ly6G$^-$Ly6C$^-$F4/80$^+$ cells), CCR2$^+$ myeloid cells (CD45$^+$CD11b$^+$CCR2$^+$ cells), CCR2$^+$ monocytes (CD45$^+$CD11b$^+$Ly6G$^-$Ly6C$^+$CCR2$^+$ cells), CCR2$^+$ macrophages (CD45$^+$CD11b$^+$Ly6G$^-$Ly6C$^-$F4/80$^+$CCR2$^+$ cells), Ki67$^+$ myeloid cells (CD45$^+$CD11b$^+$Ki67$^+$ cells), Ki67$^+$ monocytes (CD45$^+$CD11b$^+$Ly6G$^-$Ly6C$^+$Ki67$^+$ cells), TNF-α$^+$ myeloid cells (CD45$^+$CD11b$^+$TNF-α$^+$ cells), TNF-α$^+$ monocytes (CD45$^+$CD11b$^+$Ly6G$^-$Ly6C$^+$TNF-α$^+$ cells), TNF-α$^+$ macrophages (CD45$^+$CD11b$^+$Ly6G$^-$Ly6C$^-$F4/80$^+$TNF-α$^+$ cells) in blood of mice presented in Figure 5b-m, Figure 6n and Figure 7d-k.

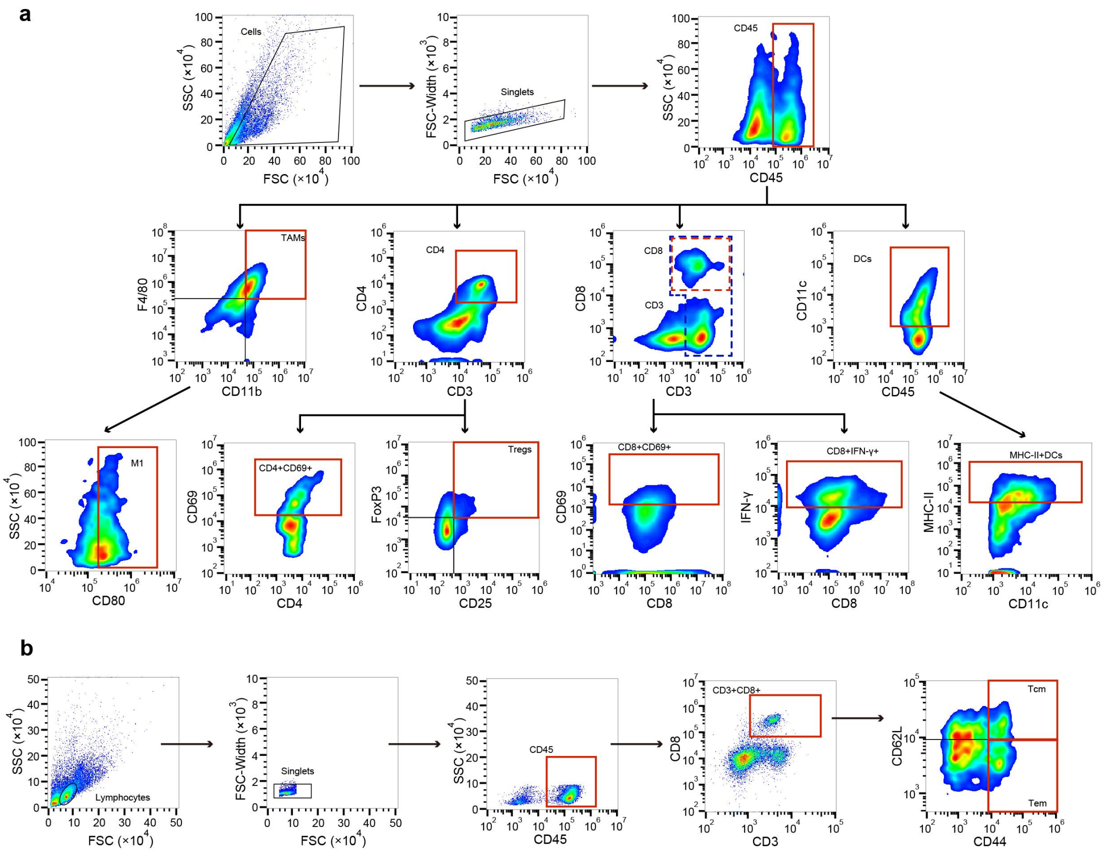

Figure S27. Gating strategies for identifying immune cells at tumor tissues, spleens and blood of tumor-bearing mice. a) Gating strategies for identifying CD3^{+} T cells (CD45^{+}CD3^{+} cells), CD4^{+} T cells (CD45^{+}CD3^{+}CD4^{+} cells), CD8^{+} T cells (CD45^{+}CD3^{+}CD8^{+} cells), CD8^{+}IFNγ^{+} T cells (CD45^{+}CD3^{+}CD8^{+}IFNγ^{+} cells), CD8^{+}CD69^{+} T cells (CD45^{+}CD3^{+}CD8^{+}CD69^{+} cells), CD4^{+}CD69^{+} T cells (CD45^{+}CD3^{+}CD4^{+}CD69^{+} cells), Tregs (CD45^{+}CD3^{+}CD4^{+}CD25^{+}FoxP3^{+} cells), M1-like TAMs (CD45^{+}CD11b^{+}F4/80^{+}CD80^{+}) and MHCII^{+} DCs (CD45^{+}CD11c^{+}MHCII^{+}) in tumor tissues of tumor-bearing mice presented in Figure 6e-m. b) Gating strategies for identifying Tcm cells (CD45^{+}CD3^{+}CD8^{+}CD44^{+}CD62L^{+} T cells) and Tem cells (CD45^{+}CD3^{+}CD8^{+}CD44^{+}CD62L^{-} T cells) in spleens and blood of tumor-bearing mice presented in Figure 6o and Figure 7l-m.

Supplemantary Table 1. Multi-color antibody combination schemes

Cell type	Antibodies	Clone	Source	Identifier
$CD80^+/CD86^+$ macrophges or DCs at injection sites	anti-mouse CD45-APC/Cy7	30-F11	BioLegend	cat. 103116
	anti-mouse CD11c-FITC	N418	BioLegend	cat. 117306
	anti-mouse CD86-APC	GL-1	BioLegend	cat. 105012
	anti-mouse CD80-PE	16-10A1	BioLegend	cat. 104707
	anti-mouse CD11b-Percp/Cy5.5	M1/70	BioLegend	cat. 101228
	anti-mouse F4/80-PE/Cy7	BM8	BioLegend	cat. 123114
$CCR2^+$/TNF-α^+ macrophges or monocytes at injection sites	anti-mouse CD45-APC/Fire 750	30-F11	BioLegend	cat. 103154
	anti-mouse CD11b-PE/Cy7	M1/70	BioLegend	cat. 101216
	anti-mouse F4/80-APC	BM8	BioLegend	cat. 123116
	anti-mouse Ly-6C-FITC	HK1.4	BioLegend	cat. 128006
	anti-mouse Ly-6G-Percp/Cy5.5	1A8	BioLegend	cat. 127616
	anti-mouse CCR2-PE	SA203G11	BioLegend	cat. 150610
	anti-mouse TNF-α-BV421	MP6-XT22	BioLegend	cat. 506328
$CD103^+$/MHC II^+ DCs in lymph nodes	anti-mouse CD45-FITC	30-F11	BioLegend	cat. 103108
	anti-mouse CD11c-PE/Cy7	N418	BioLegend	cat. 117318
	anti-mouse CD103-APC/Cy7	2E7	BioLegend	cat. 121432
	anti-mouse MHCII-Percp/Cy5.5	M5/114.15.2	BioLegend	cat. 107625
$CD80^+/CD86^+$ DCs in lymph nodes	anti-mouse CD45-APC/Cy7	30-F11	BioLegend	cat. 103116
	anti-mouse CD11c-FITC	N418	BioLegend	cat. 117306
	anti-mouse CD86-APC	GL-1	BioLegend	cat. 105012
	anti-mouse CD80-PE	16-10A1	BioLegend	cat. 104707
	anti-mouse CD11b-Percp/Cy5.5	M1/70	BioLegend	cat. 101228
H-$2k^b$-SIINFEKL DCs in lymph nodes	anti-mouse CD45-APC/Cy7	30-F11	BioLegend	cat. 103116
	anti-mouse CD11c-FITC	N418	BioLegend	cat. 117306
	anti-mouse H-2kb-SIINFEKL-PE	25-D1.16	BioLegend	cat. 141604
$CD69^+$ T cells in lymph nodes	anti-mouse CD45-FITC	I3/2.3	BioLegend	cat. 147709
	anti-mouse CD3-PE	17A2	BioLegend	cat. 100206
	anti-mouse CD4-Percp/Cy5.5	RM4-5	BioLegend	cat. 100540
	anti-mouse CD8-PE/Cy7	53-6.7	BioLegend	cat. 100722
	anti-mouse CD69-APC	H1.2F3	BioLegend	cat. 104514

CCR2+ macrophges or monocytes in blood	anti-mouse CD45-APC/Fire 750	30-F11	BioLegend	cat. 103154
	anti-mouse CD11b-PE/Cy7	M1/70	BioLegend	cat. 101216
	anti-mouse F4/80-APC	BM8	BioLegend	cat. 123116
	anti-mouse Ly-6C-FITC	HK1.4	BioLegend	cat. 128006
	anti-mouse Ly-6G-Percp/Cy5.5	1A8	BioLegend	cat. 127616
	anti-mouse CCR2-PE	SA203G11	BioLegend	cat. 150610
Ki67+ myeloid or monocytes in blood	anti-mouse CD45-FITC	30-F11	BioLegend	cat. 103108
	anti-mouse CD11b-PE/Cy7	M1/70	BioLegend	cat. 101216
	anti-mouse F4/80-BV421	BM8	BioLegend	cat. 123132
	anti-mouse Ly-6C-PE	HK1.4	BioLegend	cat. 128008
	anti-mouse Ly-6G-Percp/Cy5.5	1A8	BioLegend	cat. 127616
	anti-mouse Ki67-APC	16A8	BioLegend	cat. 652406
ex vivo blood TNF-α+ monocytes/ macrophges	anti-mouse CD45-APC/Fire 750	30-F11	BioLegend	cat. 103154
	anti-mouse CD11b-FITC	M1/70	BioLegend	cat. 101206
	anti-mouse F4/80-APC	BM8	BioLegend	cat. 123116
	anti-mouse Ly-6C-PE	HK1.4	BioLegend	cat. 128008
	anti-mouse Ly-6G-Percp/Cy5.5	1A8	BioLegend	cat. 127616
	anti-mouse TNF-α-BV421	MP6-XT22	BioLegend	cat. 506328
ex vivo CD80+ macrophages in blood	anti-mouse CD45-APC/Fire 750	30-F11	BioLegend	cat. 103154
	anti-mouse CD11b-Percp/Cy5.5	M1/70	BioLegend	cat. 101228
	anti-mouse F4/80-APC	BM8	BioLegend	cat. 123116
	anti-mouse CD80-PE	16-10A1	BioLegend	cat. 104708
CD8+ T cells in tumors	anti-mouse CD45-APC/Cy7	30-F11	BioLegend	cat. 103116
	anti-mouse CD3-FITC	17A2	BioLegend	cat. 100204
	anti-mouse CD8-PE/Cy7	53-6.7	BioLegend	cat. 100722
	anti-mouse CD69-BV421	H1.2F3	BioLegend	cat. 104527
	anti-mouse IFNγ-PE	XMG1.2	BioLegend	cat. 505808
CD4+ T cells in tumors	anti-mouse CD45-APC/Cy7	30-F11	BioLegend	cat. 103116
	anti-mouse CD3-FITC	17A2	BioLegend	cat. 100204
	anti-mouse CD4-Percp/Cy5.5	RM4-5	BioLegend	cat. 100540
	anti-mouse CD25-APC	3C7	BioLegend	cat. 101909

	anti-mouse CD69-BV421	H1.2F3	BioLegend	cat. 104527
	anti-mouse FoxP3-PE	MF-14	BioLegend	cat. 126404
TAMs in tumors	anti-mouse CD45-APC/Cy7	30-F11	BioLegend	cat. 103116
	anti-mouse CD11b-Percp/Cy5.5	M1/70	BioLegend	cat. 101228
	anti-mouse F4/80-PE/Cy7	BM8	BioLegend	cat. 123114
	anti-mouse CD80-PE	16-10A1	BioLegend	cat. 104707
DCs in tumors	anti-mouse CD45-APC/Cy7	30-F11	BioLegend	cat. 103116
	anti-mouse CD11c-FITC	N418	BioLegend	cat. 117306
	anti-mouse MHCII-Percp/Cy5.5	M5/114.15.2	BioLegend	cat. 107625
Tcm/Tem in spleens or blood	anti-mouse CD45-APC/Cy7	30-F11	BioLegend	cat. 103116
	anti-mouse CD3-FITC	17A2	BioLegend	cat. 100204
	anti-mouse CD8-PE/Cy7	53-6.7	BioLegend	cat. 100722
	anti-mouse CD44-PE	IM7	BioLegend	cat. 103024
	anti-mouse CD62L-APC	MEL-14	BioLegend	cat. 104412
